# Fine-Resolution Asymmetric Migration Estimation

**DOI:** 10.1101/2025.05.29.656894

**Published:** 2025-05-30

**Authors:** Hao Shen, John Novembre

**Affiliations:** 1 Department of Human Genetics, University of Chicago; 2 Department of Ecology and Evolution, University of Chicago

## Abstract

The genetic structure of populations is often shaped by processes and events that introduce asymmetries to gene flow between geographic locations. Existing population genetic methods like EEMS and FEEMS summarize gene flow using effective equilibrium migration rates that are assumed to be symmetric, providing representations of underlying structure that have proven useful, but that fail short of representing signatures of aysmmetric gene flow. More elaborate methods capable of inferring asymmetric gene flow exist, but are computationally intensive, restricting analyses to small sets of sub-populations (demes). Here, we introduce FRAME (Fine-Resolution Asymmetric Migration Estimation), a method that significantly reduces the computational burden of estimating asymmetric gene flow. This efficiency allows FRAME to infer asymmetric migration patterns for scenarios with a large number of demes, enabling fine-scale analyses. Under the assumed model and its equilibrium condition, FRAME’s estimates can be interpreted as backward migration rates; in non-equilibrium conditions, FRAME detects and summarizes asymmetries in gene flow with effective equilibrium migration rates, which allows a richer representation of genetic structure. We assess the method using a variety of simulated histories of gene flow and apply the method to datasets from North American gray wolves, poplar trees, white-crowned mannakin, and human archaeogenetic samples. The applications demonstrate FRAME’s scalability and flexibility in detecting complex asymmetric migration signals and population structures.

## Introduction

In spatially structured populations, migration over generations shapes genetic variation and often leads to isolation-by-distance—a phenomenon in which geographically proximal individuals are more genetically similar than those farther apart (Wright, 1943, 1946). Although widespread across species and ecosystems (Slatkin, 1985; Meirmans, 2012), this phenomenon can be complex due to spatial heterogeneity in gene flow, which causes the relationship between genetic distance and geographic distance to vary across the landscape (Bradburd and Ralph, 2019).

Many methods have been developed to reveal spatial heterogeneity in isolation-by-distance (House and Hahn, 2017; Bradburd et al., 2016; Petkova et al., 2016; Marcus et al., 2021). Of particular interest here are the methods EEMS (Petkova et al., 2016) and FEEMS (Marcus et al., 2021), which model a population in terms of a large network of connected demes at migration-drift equilibrium and infer symmetric migration rates between demes. These methods produce visual maps of regions of high/low effective migration rates, offering insights into spatial variation of gene flow.

In modeling spatial heterogeneity in isolation-by-distance, as in other areas of population genetics, a coalescent-based perspective is incredibly helpful. In particular, expected pairwise coalescent times can be used to calculate the expectations of basic summaries such as $F_{\text{ST}}$ (Slatkin, 1991), as well as in fitting models of population structure, as in EEMS or other models (Petkova et al., 2016; Lundgren and Ralph, 2019). For a meta-population composed of several sub-populations or ‘demes’, the structured coalescent (Notohara, 1990; Takahata, 1991) provides a natural framework for the computation of expected pairwise coalescence times based on backward migration and coalescence rates (Strobeck, 1987).

However, fast computation of the expected pairwise coalescent times in the structured coalescent is a major challenge. For symmetric migration, a two-pronged strategy has been employed to reduce the time complexity. The first part of the strategy is to decompose the pairwise coalescence time into the time for two lineages to enter the same deme and the time for two lineages within the same deme to coalesce (Slatkin, 1991). The second part is to approximate the expected time for two lineages to enter the same deme as one-fourth of the expected commute time of the Markov chain defined by single-lineage migration (Matsen and Wakeley, 2006). A well-established connection between circuit theory and random walks (Nash-Williams, 1959) equates expected commute times with effective resistances (i.e., resistance distances) in the corresponding electrical network, for which efficient algorithms are available (Babić et al., 2002; Bapat, 2004). This two-pronged strategy also forms the basis of the widely used ‘isolation-by-resistance’ framework (McRae, 2006; Hanks and Hooten, 2013; Dickson et al., 2018).

In the case of isotropic symmetric migration, this strategy yields exact pairwise coalescence times. Under anisotropic but still pairwise symmetric migration, it produces approximate results and underpins methods such as EEMS (Petkova et al., 2016) and FEEMS (Marcus et al., 2021).

However, many natural systems exhibit pronounced asymmetries in gene flow, which these symmetric models fail to represent. Asymmetric migration can arise from persistent directional forces such as ocean currents (White et al., 2010; Bertola et al., 2020), prevailing winds (Kling and Ackerly, 2021), or river networks (Alp et al., 2012; Thomaz et al., 2016), as well as from historical demographic events such as range expansions (Ramachandran et al., 2005; Excoffier et al., 2009) and mass migrations (Haak et al., 2015; Lipson et al., 2017). Accurately modeling such asymmetries is crucial for reconstructing spatial population histories.

From a computational standpoint, accounting for asymmetry introduces significant challenges. As pointed out by Lundgren and Ralph (2019), approximate strategies that perform well under symmetric assumptions may lead to substantial distortions when applied to asymmetric settings. To address this issue, Lundgren and Ralph proposed directly solving the matrix equation for expected pairwise coalescent times—originally introduced by Strobeck (1987)—by vectorizing it into a system of linear equations. While this approach provides exact solutions, its computational complexity scales as $O\left(d^{6}\right)$, where d is the number of demes, making it impractical for large-scale applications.

Here, we introduce FRAME (Fine-Resolution Asymmetric Migration Estimation). FRAME employs techniques from computational linear algebra and solves the structured coalescent equation with time complexity of $O\left(d^{4}\right)$. By integrating this approach with the efficient gradient-based optimization and penalizedlikelihood in FEEMS, FRAME enables the inference of fine-resolution asymmetric migration patterns with significantly improved computational efficiency.

## Results

### Model specification and inference strategy

We model population structure using a weighted directed graph, where vertices represent demes and edges connect demes connected by migration. A directed edge from deme i to deme j is assigned a weight $m_{ij}$, which biologically represents the rate at which individuals in deme i have ancestry in deme j. From the structured coalescent perspective, $m_{ij}$ is the (backward) migration rate of lineages from deme i to deme j.

These migration rates form the migration rate matrix M, which is also understandable as the weighted adjacency matrix of the graph, and which encapsulates all the parameters regarding gene exchange between demes. The Laplacian of the graph can then be computed as L=D−M, where D is a diagonal matrix with its $(i,i{)}_{\text{th}}$ entry being the sum of the $i_{\text{th}}$ row of M. Backward migration in the structured coalescent corresponds to a jump process on the graph, with −L serving as the transition-rate matrix.

We parameterize the coalescence rate per deme by $\gamma =\left(\gamma_{1},\gamma_{2},\ldots ,\gamma_{d}\right)$, where $\gamma_{i}$ represents the coalescence rate in deme i. The equation for expected pairwise coalescence times can then be computed from L and γ (Strobeck, 1987)

(1) $$\text{diag}\left\{\gamma \right\}\text{diag}\left\{T\right\}+LT+TL^{T}=1_{d\times d}.$$

Here T is the matrix such that its $(i,j{)}_{\text{th}}$ entry $T_{ij}$ represents the expected coalescence time between two randomly chosen haploid samples, one from deme i and another from deme $j,1_{d\times d}$ is a matrix of all ones.

Solving this equation using a direct vectorized methods is computationally expensive: solving a linear system with $O\left(d^{2}\right)$ unknowns has a complexity of $O\left(d^{6}\right)$. This computational burden limits the application of existing methods to large datasets.

**Figure 1:**
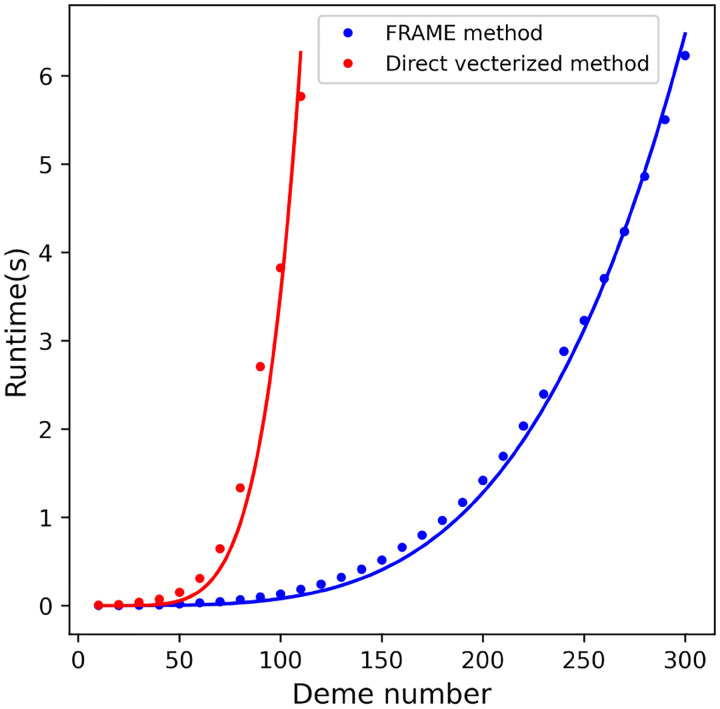
Runtimes for FRAME method vs the direct solution of the linear system of Equation 1. Average runtimes for solving equation (1) using FRAME and brute-force methods are shown, for a given number of demes (x-axis) connected via random migration rates in a fully connected graph and with random coalescence rates (see Methods).

To overcome this limitation, we develop a method that solves it with a complexity of $O\left(d^{4}\right)$, enabling larger-scale inference and finer spatial resolution (Methods and Supplementary Note). To do so, we use techniques from computational linear algebra and exploit the structural similarity between this equation and the Lyapunov equation. In Fig. 1, we compare the speed of the method to a direct vectorized method optimized by sparse techniques, demonstrating that our method is significantly faster in computation. For example, it solves equation (1) with 100 demes in an average of 0.13 seconds, whereas the optimized direct vectorized method takes an average of 3.82 seconds.

Building upon this computational advance, we develop the inference machinery of FRAME. Detailed computational steps are provided in the Methods section and Supplementary Note. Here, we briefly outline the logic and key steps.

An important aspect about the parameterization is the relationship between γ and M. If population sizes are fully determined by migration,the jump process specified by M implies a corresponding stationary probability distribution vector $\pi =\left(\pi_{1},\pi_{2},\ldots ,\pi_{d}\right)$. In such a case, if we denote the total population size by $N_{T}$, the population size of deme i can be expressed as $N_{T}\pi_{i}$, and $\gamma_{i}$ would be $\frac{1}{N_{T}\pi_{i}}$ (Strobeck, 1987). However, such a model can be unrealistic in many cases, for example, when migration occurs through gametes, and population sizes are determined by ecological capacity rather than the movement of individuals.

One way to relax the relationship between coalescence rates and the stationary distribution is to set coalescence rates as free parameters. However, this approach increases the risk of identifiability issues. An alternative is to assume all coalescence rates are equal, yet this constrains the model’s unrealistically. To address this, we adopt a compromise by set $\gamma_{i}=\frac{c}{\pi_{i}^{\alpha }}$, where c is scaling constant greater than 0, and α is a constant between 0 and 1. When α is 0, the model assumes all coalescence rates are equal. When α is 1, the model corresponds to the case where coalescence rates γ are fully determined by the stationary distribution π implied by M (in which case c is interpretable as $\frac{1}{N_{T}}$).

Similar to FEEMS, FRAME uses a penalized maximum likelihood approach to estimate M,c and α. That is, we minimize the objective function l(M,c,α)+λΨ(M) where .l(M,c,α)=−logP(D∣M,c,α) denotes the negative log likelihood of the parameters given the observed genetic distances, Ψ(M) represents a penalty function that favors smoothness in the inferred weight parameters, and λ is a scalar multiplier of the penalty function. For the penalty function, FRAME employs a formulation that slightly differs from that of FEEMS (Methods and Supplementary Note). For fixed values of λ we solve the optimization problem using the L-BFGS (Byrd et al., 1995) algorithm, and then we use cross-validation based on the model’s prediction of allele frequencies to choose λ.

### Example outputs

**Figure 2:**
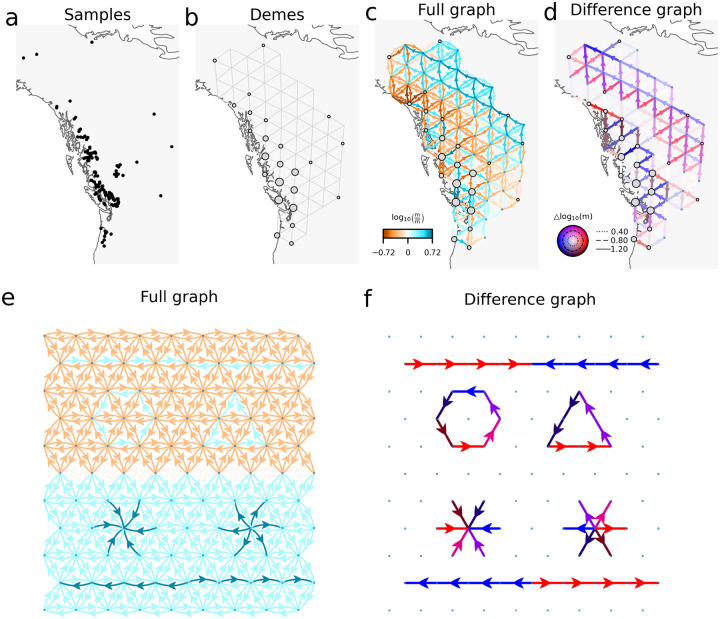
Introduction to FRAME with example outputs. **(a-d)** A schematic overview of FRAME using the poplars dataset. **(a)** The input consists of sample genotypes and their spatial positions. **(b)** FRAME constructs demes and assigns samples to their nearest demes. **(c)** The full graph representation of FRAME fit, displaying all edges. **(d)** The difference graph representation of FRAME fit, emphasizing asymmetries. **(e-f)** Various migration patterns represented by FRAME, including spatially converging lineages, spatially diverging lineages, cyclic rotating lineages, and directionally migrating lineages.

Figure 2 (a–d) illustrates an application of FRAME using a dataset of two closely related poplar species (*Populus trichocarpa* and *Populus balsamifera*) analyzed previously by Lundgren and Ralph (2019). The input data consists of the latitudes, longitudes, and genotypes of sampled individuals. FRAME constructs a network of demes (detailed in Methods) to model spatial genetic structure and assigns samples to nearby demes based on proximity.

The ‘full graph’ (Figure 2c) provides an overview of all inferred migration rates across the landscape, capturing both the magnitude and direction of gene flow. In contrast, the ‘difference graph’ (Figure 2d) highlights migration asymmetries by calculating the difference in log-scaled migration rates between connected demes, revealing more clearly directional biases in gene flow backward in time. (Additional possible summary graphs and related demographic parameters for the empirical anlyses that follow are presented in the Supplementary Notes, Supplementary Figs. 7–14.)

Since the graph edges represent backward migration of lineages, the patterns can be naturally interpreted in the context of the structured coalescent. Several typical patterns are shown in Figure 2 (e–f). One pattern is where arrows point from surrounding demes to a central deme. This indicates that lineages from surrounding demes migrate backward to the center at higher rates than they migrate away from it. We call this a pattern of ‘spatially converging lineages’. Conversely, a pattern of ‘spatially diverging lineages’ is a pattern where lineages in the central deme migrate backward to the surrounding demes at a higher rate than they migrate to it, suggesting hybridization/admixture-like dynamics are likely to have occurred at the center. Patterns where lineages migrate backward along a path in a certain direction at higher rates represent what we term ‘directionally migrating lineages’. Cycles on the graph are also theoretically possible and for shorthand we call these patterns of ‘cyclic rotating lineages’.

### Simulation

**Figure 3:**
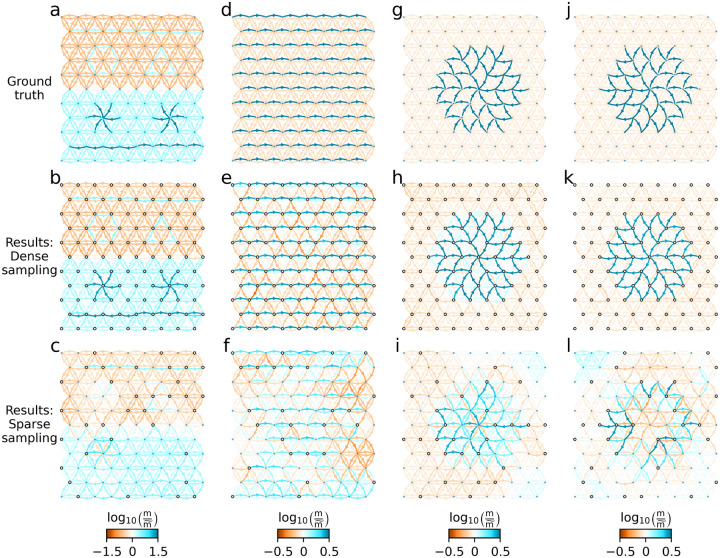
FRAME fit to equilibrium migration simulations with different patterns and sampling schemes The first column (**a–c**) presents the ground truth and FRAME fit to coalescent simulations featuring small-scale migration patterns. The second column (**d–f**) shows results for simulations with a large scale pattern of directionally migrating lineages (‘west’ to ‘east’ here). The third column (**g–i**) illustrates cases with a large scale patterns of spatially converging lineages, while the final column (**j**–**l**) represents scenarios with a large scale pattern of spatially diverging lineages (For details of the simulations, see Methods).

Figure 3 illustrates FRAME’s performance under the equilibrium assumption in a standard stepping stone model with asymmetric migration. The columns each contain results for a different migration scenario. The first column shows results for the combination of small scale patterns mentioned above (Fig. 1e,f). The next three columns show a large scale pattern of directionally migrating lineages (second column), spatially converging lineages (third column) and spatially diverging lineages (last column). The corresponding difference graphs are shown in Supplementary Fig. 1.

When sampling is dense and the dataset is large (Figure 3, panels b to k), both small-scale patterns and large-scale patterns are faithfully recovered, closely matching the ground truth. In contrast, when sampling is sparse (Figure 3, panels c to l), with fewer demes containing observed samples, FRAME’s ability to detect migration patterns depends on the scale of the patterns. Large scale patterns are more readily detected even with sparse sampling. However, small scale patterns are often missed or underrepresented due to the lack of samples near regions where these patterns occur. Under sparse sampling, the inferred networks recover broader migration trends, while finer details are smoothed out or lost. Fig 3g–i and j–l are interesting to contrast, in that the broad structure of the asymmetries is similar in all but direction. The inference results show more fidelity at recovering the true migration parameters for the case of backwards-in-time spatially converging lineages (Figure 3, panels g to i) vs for the diverging case (Figure 3, panels j-l).

**Figure 4:**
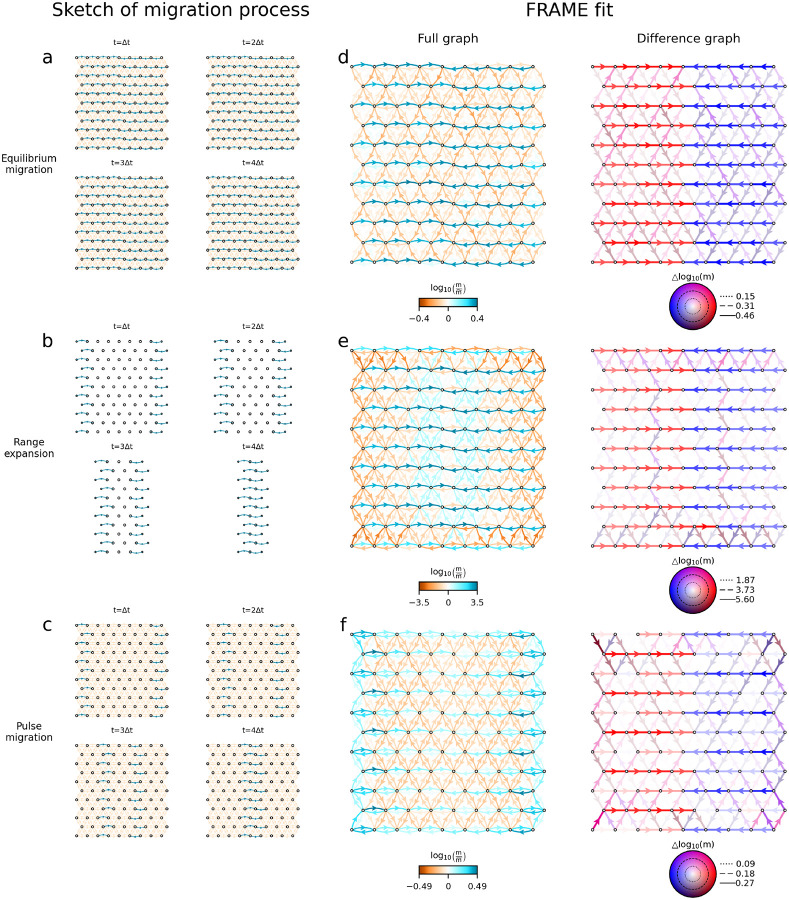
Simulations demonstrate FRAME’s ability to detect asymmetric migration signals even under non-equilibrium conditions **(a–c)** Illustrations of the simulated migration scenarios (backward in time). **(a)** Equilibrium migration. **(b)** Range expansion. **(c)** Sequential pulse migration. (**d–f**) FRAME’s fit to the corresponding migration settings.

All simulations in Figure 3 assume equal population sizes across demes. In supplementary Figs. 2 and 3 we demonstrate that FRAME can also effectively recover migration patterns when population sizes are proportional to the stationary distribution implied by the migration matrix. To further evaluate the method’s performance, we extended the simulations to non-equilibrium conditions, as illustrated in Figure 4 (see Methods and Supplementary Note for detailed parameter settings). The first row of Fig. 4 illustrates a stepping-stone model with directionally migrating lineages from the left and right boundary to the center.

The second row illustrates a range expansion scenario. Forward in time, this corresponds to a classical range expansion, where migrants originating from the central line progressively occupy new demes and undergo exponential growth until reaching carrying capacity. Migration in this scenario is strictly one-way, with no movement occurring from newly colonized demes back to the original central demes.

The third row represents a scenario of sequential pulse migration. Forward in time, this corresponds to a wave of asymmetric migration propagating outward from the central line toward both edges, eventually reaching the boundary, added upon a background of steady-state symmetric migration rate between neighboring demes.

In all three scenarios, FRAME successfully detects signals of directional gene flow and the inferred migration networks reflect the underlying patterns of backward-in-time gene flow. As a point of caution though, the three unique demographic histories all result in difference graphs that are qualitatively similar, highlighting how interpretation of results from real data requires considering multiple possible generating processes.

### Application to empirical datasets

**Figure 5:**
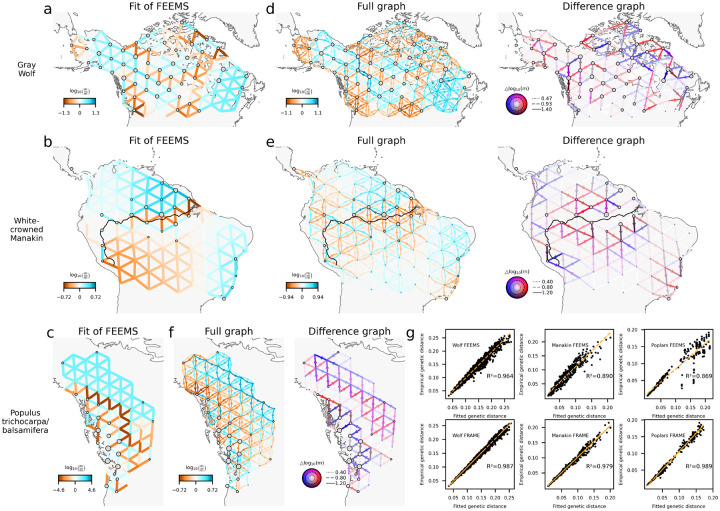
Comparison of FRAME with FEEMS on empirical datasets. **(a–c)** FEEMS fit on North American gray wolf, white-crowned manakin, and *Populus trichocarpa*/*balsamifera* datasets **(d–f)** FRAME fit on North American gray wolf, white-crowned manakin, and *Populus trichocarpa*/*balsamifera* datasets **(g)** Model fit comparison between FRAME and FEEMS.

Figure 5 shows the application of FRAME to North American gray wolves (Schweizer et al., 2016; Shastry et al., 2025), white-crowned manakins (Berv et al., 2021), and two poplar species (*Populus trichocarpa*/*balsamifera*) (Geraldes et al., 2014), with a comparison to FEEMS.

In the gray wolf dataset, FEEMS already captures migration structure and connectivity well. FRAME introduces slight improvement ($R^{2}$ from 0.964 to 0.987) by accounting for asymmetries in migration, suggesting that symmetric migration remains a reasonable approximation for this dataset.

In the white-crowned manakin dataset, FRAME shows a more substantial improvement over FEEMS ($R^{2}$ from 0.890 to 0.979). Both FEEMS and FRAME identify reduced gene flow along the Amazon River, consistent with results from EEMS in prior research (Berv et al., 2021). The difference graph highlights multiple locations with spatially converging lineages, which correspond to populations identified in earlier studies as having minimal levels of admixture. Additionally, FRAME detects signals of backwards directionally migrating lineages from Western Napo towards northwest Amazonia and the Madeira River, supporting the interpretation of Western Napo as an area receiving ancestries from both northern and southern populations. This aligns with previous findings of introgression from northern populations into Western Napo.

The dataset of poplars exhibits the largest improvement when comparing FRAME to FEEMS ($R^{2}$ from 0.869 to 0.989). FRAME captures clear asymmetries, with a pattern of directionally migrating lineages from the southeast to the northwest (note the high gene flow along edges oriented from southeast to northwest along the northeastern boundary of the modeled region). Additionally, locations with spatially converging lineages and spatially diverging lineages identified in the middle-west region align with the known ecological and historical patterns of the species (Lundgren and Ralph, 2019; Geraldes et al., 2014).

We also compare FRAME to the method introduced by Lundgren and Ralph (2019) using the poplars dataset, as shown in Supplementary Fig. 4. For the coarse resolution model with 9 demes, FRAME produces results comparable to Lundgren’s method with less computation time (Fig. 1).

Given that many asymmetric migration patterns have been observed in ancient human DNA datasets from Europe (e.g. Lipson et al., 2017; Olalde et al., 2019; Allentoft et al., 2015; Haak et al., 2015), we applied FRAME to assess its ability to recover such patterns. We analyzed ancient DNA data from the Allen Ancient DNA Resource (Mallick et al., 2024), processed following the filtering procedures described in the Supplementary Note. Our analyses focused on two broad time periods: (1) 7000–3500 BCE, corresponding to the Neolithic expansion across Europe (Lipson et al., 2017; Olalde et al., 2019); and (2) 3500–1500 BCE, marked by the spread of genetic ancestry associated with Steppe pastoralists (Allentoft et al., 2015; Haak et al., 2015). Although the coalescent model underlying FRAME assumes contemporaneous sampling, we used broad time windows to ensure sufficient sample sizes. For comparison, we also examined narrower intervals (4500–3500 BCE and 2500–1500 BCE; see Supplementary Figures 5 and 6).

**Figure 6:**
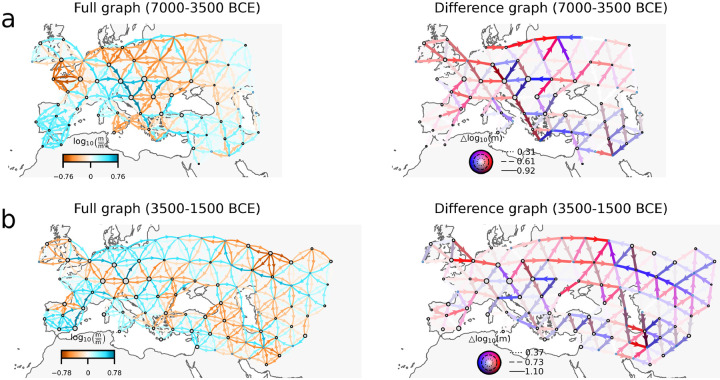
Application of FRAME to aDNA datasets **(a)** FRAME analysis of Eurasian human populations from 7000–3500 BCE (Neolithic expansion) **(b)** FRAME analysis of Eurasian human populations from 3500–1500 BCE (Steppe expansion).

For the 7000–3500 BCE dataset, the difference graph directionally migrating lineages aligned in paths leading towards the area of Anatolia: one path begins in the British Isles and continues southeast; another originates in central-western France and moves eastward; and a third starts in northeast Europe and points southwest. These paths align to a degree with the observed spread of the Linear Pottery Culture (LBK) cultures (Lipson et al., 2017). We also observe directionally migrating lineages aligned in a path that moves northeast along the Northern Mediterranean coastline and towards Anatolia, aligning with the proposed Mediterranean route of Neolithic expansion (Olalde et al., 2019). These results appear consistent with Anatolia as the geographic origin of farmer groups associated with the Mediterranean Cardial and LBK cultures (Olalde et al., 2015). The difference graph (Figure 6a, right panel) also shows directionally migrating lineages aligned along a path northeastward out of present-day Romania, arcing westward to Central Europe, and looping back to the area of the Balkans. This trajectory may reflect gene flow processes shaped by the Carpathian Mountains, a potential moderator of gene flow suggested by the full graph.

For the 3500–1500 BCE dataset, the difference graph (Figure 6b, right panel) highlights strong signals of directionally migrating lineages from Central and Eastern Europe to the Pontic-Caspian steppe, aligning with the westward movement of Yamnaya-related populations (Haak et al., 2015). In the opposite direction, FRAME also detects directionally migrating lineages from Central Asia extending westward to the Pontic-Caspian steppe. This branch of ancestral flow potentially corresponds to movements associated with the Sintashta and Andronovo cultures (Allentoft et al., 2015).

The smaller time window analyses do not fully replicate the features observed in the broader windows, likely due to reduced sample sizes or time-varying gene flow dynamics during these periods. Comparing the 7000–3500 BCE and 4500–3500 BCE analyses (Supplementary Fig. 5), we observe some shared asymmetries but also notable differences—for example along the Mediterranean, where the narrower window lacks samples from southern France. The 3500–1500 BCE and 2500–1500 BCE analyses (Supplementary Fig. 6) show more agreement, with the asymmetries plausibly reflecting gene flow out of the Steppe region being a shared signal in both analyses.

## Discussion

FRAME is a method for analyzing spatial population structure, aimed at detecting fine-scale asymmetric migration patterns. It offers a way to visualize and quantify directional gene flow, extending beyond existing symmetric models to provide additional perspectives on population dynamics. With a focus on flexibility and computational efficiency, FRAME is designed to handle large, fine-resolution datasets, supporting the study of potentially complex population structure.

In our application of the method to four species, we found that the symmetric migration model works comparatively well for the North American gray wolf dataset, while the poplar, mannakin, and ancient human datasets exhibit strong signals of asymmetric gene flow. These findings underscore the importance of modeling asymmetry when reconstructing migration histories. Applying FRAME to additional species may help identify how widespread signatures of asymmetric gene flow are and what ecological or evolutionary factors drive explain them.

While FRAME performs well in both simulations and empirical datasets, several important considerations should be kept in mind when applying the method. First, although FRAME is designed to handle large datasets, the spatial resolution it can manage is lower than methods like FEEMS, which can accommodate thousands of demes. Consequently, FRAME should not generally be viewed as an approximation to a continuous spatial surface. Decisions about whether to explicitly represent internal habitat discontinuities, such as lakes or mountains, should depend on the study’s specific context. At coarser resolutions, the placement of network nodes can affect the results, and adjustments guided by biological insights may be prudent.

Second, because FRAME assumes equilibrium conditions, it has limited capacity to explicitly model temporally varying migration dynamics. By averaging genomic signals over the coalescent timescale, FRAME simplifies calculations, but as a result it cannot capture detailed temporal changes in migration patterns. Consequently, FRAME’s results depend significantly on the timing of past migration events. For example, if a population transitions from migration pattern A to pattern B, samples collected shortly after this transition may predominantly reflect the earlier pattern, whereas later samples would reflect the new equilibrium. This temporal dependency explains why FRAME is less suited for detecting deep, continent-scale radiations—such as the proposed radiation out of the Andes observed in the white-crowned manakin dataset(Berv et al., 2021)—which are better analyzed using complementary phylogenetic approaches.

Furthermore, migration events often vary in timing and duration across different regions, causing local signals to emerge and fade asynchronously. Because FRAME summarizes these genomic signals into averaged migration patterns, it is less suited for precisely resolving event sequences in non-equilibrium scenarios. For instance, in a scenario where deme B receives migrants from both demes A and C, FRAME may identify B as a zone of spatially diverging lineages but cannot reveal whether migration from A preceded migration from C or vice versa. Similarly, when we observe a directionally migrating lineage in the difference graph, say from deme A to B to C, this alone does not imply that, forward in time, the migration from C to B precedes the migration from B to A. In these cases, the compatibility between FRAME results and non-equilibrium migration scenarios following a given temporal ordering provides a necessary condition for such interpretations, but is not sufficient to confirm them.

These considerations emphasize the importance of careful dataset construction and cautious interpretation of results. While FRAME effectively identifies fine-scale migration patterns, its equilibrium-based approach constrains its ability to reconstruct deeper evolutionary histories.

To improve the reconstruction of non-equilibrium dynamics, two possible future directions are: 1) develop methods that analyze time series data; 2) integrate haplotype-based information, akin to how the MAPS method (Al-Asadi et al., 2019) uses the length distribution of ‘long pairwise shared coalescence segments’ (also known as ‘identity by descent’ tracts). While either approach would require solving additional modeling and computational challenges, it would move the field closer to an ideal method that could reconstruct high-resolution spatiotemporal maps of gene flow.

## Methods

### The expected pairwise coalescence times

The matrix T, whose $(i,j{)}_{\text{th}}$ entry represents the expected coalescence time between an haploid individual from deme i and another from deme j, given the migration rates and coalescence rates, can be solved using equation (1). However, solving this equation with a direct vectorized approach has time complexity $O\left(d^{6}\right)$, which is computationally prohibitive for fine-resolution analyses. We reduce the computational complexity by leveraging the similarity between this equation and continuous-time Lyapunov equations (Gajic and Qureshi, 2008). In short, let G be an arbitrary symmetric matrix, we solve the more general equation

(2) $$\text{diag}\left\{\gamma \right\}\text{diag}\left\{X\right\}+LX+XL^{T}=G,$$

by solving a series of simpler singular continuous-time Lyapunov equations. $LX+XL^{T}=G$ and $LX+XL^{T}=E_{i},i=1,2,\ldots ,d$ in the least square sense, where $E_{i}$ is the matrix with its $(i,i{)}_{\text{th}}$ entry equal to 1 and all other entries equal to 0. The solution can then be expressed as a linear combination of the solutions to these simpler equations, along with some constants. The coefficients of this linear combination can be determined either using the method of undetermined coefficients or through a step-by-step low-rank update approach, which offers greater computational robustness. This approach reduces the complexity to $O\left(d^{4}\right)$. The detailed algorithm is provided in the Supplementary Note.

### The penalized likelihood

Assume we have o observed sample groups with corresponding o×d assignment matrix J if sample group i is in deme $d_{i},J_{id_{i}}=1$, otherwise $J_{id_{i}}=0$. Assume in sample group, we have $n_{i}$ copies of haploid data on p SNPs (every diploid counts as two copies). Let allele state of sample group i, locus j, copy k be $Z_{ijk}$ (coded as 0 or 1), and let $f_{ij}=\frac{\sum_{k=1}^{n_{i}}Z_{ijk}}{n_{i}}$ be the allele frequency at sample group i, locus j, we can define vectors $f_{j}={\left(f_{1j},\ldots ,f_{oj}\right)}^{T},j=1,2,\ldots ,p$. Each vector $f_{j}$ can be viewed as an independent realization of the random vector of allele frequencies assuming perfect segregation (justified in the Supplementary Note). Let $T_{\text{mrca}}$ and $T_{\text{tot}}$ be the expected height and expected total branch length of the coalescent tree across all samples; K be a o×1 vector such that its $i_{\text{th}}$ entry is $\frac{1}{n_{i}}$, the mean of the random vector of allele frequencies is then $\frac{T_{\text{mrca}}}{T_{\text{tot}}}\times 1_{o\times 1}$ and the covariance matrix is given by (Supplementary Note)

(3) $$\Sigma =\sigma^{2}\left(1_{o\times o}-\rho \underline{T}\right),$$

where $\sigma^{2}=\frac{T_{\text{mrca}}}{T_{\text{tot}}}\left(1-\frac{T_{\text{mrca}}}{T_{\text{tot}}}\right),\rho =\frac{1}{T_{\text{mrca}}\left(1-\frac{T_{\text{mrca}}}{T_{\text{tot}}}\right)}$ and $\underline{T}=JTJ^{T}-\text{diag}\left\{JTJ^{T}\right\}\text{diag}\{K\}$.

To remove the mean and focus on the covariance structure, we apply a contrast matrix $C\in R^{o-1\times o}$ (Supplementary Note). Assume each $f_{j}$ obeys a multivariate normal distribution, we then have

(4) $$Cf_{j}\sim N\left(0,C\Sigma C^{T}\right).$$

The sample covariance matrix $\hat{\Sigma }$ after the transformation obeys a Wishart distribution

(5) $$C\hat{\Sigma }C^{T}\sim W_{o-1}\left(\frac{C\Sigma C^{T}}{p},p\right).$$

Notice that the transformed covariance matrix can be expressed as

(6) $$C\Sigma C^{T}=-\frac{1}{T_{\text{tot}}}C\underline{T}C^{T},$$

where $T_{\text{tot}}$, the expected total coalescence time, acts as a nuisance parameter. $T_{\text{tot}}$ is a complex function of the underlying model and the sample configuration across demes, making it challenging to disentangle directly during inference.

To address this, we absorb it into other parameters by redefining $L^{\prime }=T_{\text{tot}}L;\;\gamma^{\prime }=T_{\text{tot}}\gamma$, and $T^{\prime }=\frac{T}{T_{\text{tot}}}$. This absorption simplifies the parameterization and can be justified within the maximum likelihood framework (see Supplementary Note). Importantly, the structured coalescent equation remains valid under this rescaling. Thus, we can drop the primes and proceed by treating $L^{\prime },\;\gamma^{\prime },\;T^{\prime }$ as the new L,γ,T, respectively.

After renormalizing the time scale and removing constants, the negative log-likelihood is expressed as:

(7) $$l=\frac{p}{2}\left\{-\text{tr}\left[{\left(C\underline{T}C^{T}\right)}^{-1}C_{\text{ob}}\hat{\Sigma }C^{T}\right]+\text{log}\left|-\left(C\underline{T}C^{T}\right)\right|-\text{log}\left|C\hat{\Sigma }C^{T}\right|\right\}.$$

Let $\Omega =\left\{(i,j)|m_{ij}>0\right\}$ represent the edge set, and let $\Omega_{i}=\{(i,t)\in W\}\cup \{(s,i)\in \Omega \}$ denote the edges connected to node i. Then, the penalty can be written as λΨ, where

(8) $$\Psi =\frac{1}{2}\sum_{i=1}^{d}\sum_{\begin{array}{c} \left(i_{1},j_{1}\right)\in \Omega_{i},\\ \left(i_{2},j_{2}\right)\in \Omega_{i} \end{array}}\frac{1}{{\left|\Omega_{i}\right|}^{2}}\left[{\left(\frac{m_{i_{1}j_{1}}}{\tilde{m}}-\frac{m_{i_{2}j_{2}}}{\tilde{m}}\right)}^{2}+{\left(\frac{\tilde{m}}{m_{i_{1}j_{1}}}-\frac{\tilde{m}}{m_{i_{2}j_{2}}}\right)}^{2}\right],$$

and $\tilde{m}$ is the geometric mean of all the nonzero migration rates.

This penalty function controls the smoothness of the fitting and penalizes both low and high migration rates equally, preventing ill-conditioned situations where parameters vary across many orders of magnitude.

The objective function is then l+λΨ, with l defined in 7 and Ψ defined in equation (8). The optimization problem then is to find parameters $\hat{M},\hat{c},\hat{\alpha }$ that minimize l+λΨ.

### Optimization

We infer the migration rates matrix $\hat{M}$ by solving the following optimization problem:

(9) $$\hat{M},\hat{c},\hat{\alpha }=\underset{(M,c,\alpha )\in 𝒟}{\text{argmin}}(l+\lambda \Psi )$$

where $𝒟={𝒟}_{ℳ}\times R^{+}\times [0,1]$, ${𝒟}_{ℳ}=\left\{M|m_{ij}>0\;\text{if}\;(i,j)\in \Omega ,m_{ij}=0\;\text{if}\;(i,j)\notin \Omega \right\}$.

Similarly to FEEMS, we solve the optimization using the L-BFGS method (Byrd et al., 1995). A crucial step in this optimization is the efficient computation of the gradient, which requires solving equations of the form (Supplementary Note):

(10) $$\text{diag}\left\{\gamma \right\}\text{diag}\left\{X\right\}+L^{T}X+XL=G,$$

where G is an arbitrary symmetric matrix. Although this equation is different from equation (2), the computational techniques used to solve (2) can be applied here with appropriate modifications, as detailed in the Supplementary Note. The time complexity of computing the gradient remains $O\left(d^{4}\right)$.

### Cross-validation

The observed demes are split into a training set and a testing set. For each candidate value of λ, we fit the model on the training set and predict allele frequencies (after contrasting) for the testing set. Specifically, let $f_{j}^{c}=Cf_{j}$ and $\Sigma^{c}=C\Sigma C^{T}$. Then:

(11) fjc=fjc,trainfjc,validation~N0,Σc,trainΣc,covΣc,covΣc,validation.

Prediction can then be made using:

(12) $$E\left[f_{j}^{c,\text{validation}}|f_{j}^{c,\text{train}}\right]=\Sigma^{c,\text{cov}}{\left(\Sigma^{c,\text{train}}\right)}^{⊤}f_{j}^{c,\text{train}}.$$

The value of λ that minimizes the prediction error is selected, and the model is then refit to the entire dataset using this optimal λ.

Leaving one deme out at a time for cross-validation is the default, though for datasets with a large number of observed demes, using 10-fold cross-validation can improve efficiency.

### Simulations

We utilize msprime (Baumdicker et al., 2022) to simulate the structured coalescence process for each SNP. This procedure generates a dataset containing 100, 000 simulated SNPs for all sampled individuals. In the case of dense sampling, individuals are selected from every deme, whereas for sparse sampling, each deme is chosen with a probability of 0.25. When a deme is selected, 10 individuals are sampled from it.

In all large-scale pattern simulations shown in Figure. 3 (d–l), the ground truth graph uses deep blue edges to represent migration rates that are 3 times greater than the baseline $m_{\text{base}}$, which is depicted with shallow orange edges. We set the baseline migration rates and local population sizes (N) so that $Nm_{\text{base}}=0.1$.

In the small-scale pattern simulation shown in Figure. 3 (a–c), assuming the base migration rate (i.e., the migration rate of the edges in the middle row) is 1, the edges in the lower half of the graph have base migration rates of 3, while the edges in the upper half have base migration rates of 0.3. Additionally, all patterns exhibit migration rates that are 10 times higher than the surrounding base migration rates. Specifically, the patterns in the upper half of the graph will have a migration rate of 3, while patterns in the lower half will have migration rates of 30. This configuration ensures that all patterns are clearly distinguishable from the background while also maintaining geographic differentiation within the background itself.

The setting for non-equilibrium simulations are more complex and are detailed in the Supplementary Note.

### Empirical data sets

We applied FRAME to following datasets: a North American gray wolve dataset with 108 individuals and 17, 729 snps, a poplars dataset with 431 individuals and 30, 756 snps, a white-crowned manakin dataset with 233 individuals and 1960 snps, a 7000–3500 BCE ancient human dataset with 975 individuals and 92, 967 snps, a 3500–1500 BCE ancient human dataset with 1361 individuals and 51, 306 snps, a 4500–3500 BCE ancient human dataset with 383 individuals and 204, 791 snps and a 2500–1500 BCE dataset with 916 individuals and 48, 553 snps. The assessments first three datasets were primarily based on previous studies (Marcus et al., 2021; Shastry et al., 2025; Geraldes et al., 2014; Berv et al., 2021) with missing data imputed using the allele frequencies. The filtering procedures for the ancient human datasets were more complex, given the low genotype rates typical of aDNA samples. Details about the datasets are provided in the Supplementary Notes.

### Extended Graph Representations

Beyond the primary representations—the full graph and difference graph—two other options for visualizing the migration dynamics are what we term a ‘base graph’ and ‘summary graph’ (see Supplementary Fig. 7–14).

Supplementary Fig. 7 presents these additional visualizations. The ‘base graph’ illustrates mean migration rates between connected demes on a log scale, providing a complementary perspective to the difference graph. The qualitative similarity between the base graph and FEEMS outputs highlights the broader relationship between these models. The ‘summary graph’ aggregates backward migration rates at each node into a vector, summarizing overall trends in directionally migrating lineages.

Together, these representations enhance the interpretability of migration patterns and offer diverse tools for exploring population structure. A comprehensive set of visualizations, including all four graph representations and related demographic parameters for the empirical datasets, is presented in Supplementary Figs. 8–14. Notably, these figures all show that coalescence rates are inferred as relatively uniform rather than inversely proportional to the stationary distribution, suggesting that local effective population sizes in these datasets may be largely independent of migration.

### Network Construction

FRAME leverages an automatic method to construct the network similar to that used in FEEMS (Marcus et al., 2021), which generates a dense triangular lattice over the study region. We define the outer boundary using the convex hull of sample locations with a layer of buffer, which is manually refined to align with the species’ habitat and relevant geographic features. To ensure uniform spatial coverage, we intersect the boundary with a precomputed global triangular grid projected onto the Earth’s surface (Sahr et al., 2003). Multiple grid resolutions are available, allowing users to evaluate model performance across different spatial scales. Alternatively, users can manually design the network and assign samples to demes using empirical knowledge.

## Supplementary Material

Supplement 1
