# Supplementary material for: Fine-Resolution Asymmetric Migration Estimation
Source: bioRxiv. 2025 May 30:2025.05.29.656894. Preprint. [Version 1] doi: 10.1101/2025.05.29.656894 (PMC12154593; doi:10.1101/2025.05.29.656894)
Supplement: Supplement 1 [file media-1.pdf]

Fine-Resolution Asymmetric Migration Estimation

Hao Shen[1] and John Novembre[1,2]

[1]Department of Human Genetics, University of Chicago
[2]Department of Ecology and Evolution, University of Chicago

May 29, 2025

Supplementary Note

1 Mathematical details of the FRAME method **2**
1.1 Overview 2
1.2 Covariance matrix of allele frequencies 3
1.3 The penalized likelihood function 4
1.4 Computing of the expected pairwise coalescence time 7
1.5 Computing of the gradient 14

2 Settings of simulations and empirical datasets **20**
2.1 Non-equilibrium simulations 20
2.2 Empirical datasets 21

1 Mathematical details of the FRAME method

1.1 Overview

The structured coalescent process combines backward migration among demes and coalescence events when lineages co-occur in the same deme. This process canbe modeled in terms of a weighted directed graph. The nodes of the graph are denoted by $\{1, 2, ..., d\}$, each representing a deme. The weight associated with the edge from node i to node j is m_{ij}, representing the backward migration rate from i to j. Biologically, m_{ij} is the rate at which deme i receives ancestry from deme j. The Laplacian of the weighted directed graph is then

$$L = \begin{pmatrix} \sum\limits_{i\neq 1} m_{1i} & -m_{12} & \dots & -m_{1d} \\ -m_{21} & \sum\limits_{i\neq 2} m_{2i} & \dots & -m_{2d} \\ \vdots & \vdots & \dots & \vdots \\ -m_{d1} & -m_{d2} & \dots & \sum\limits_{i\neq d} m_{di} \end{pmatrix}.$$

The transition rate matrix of the jump process is defined as $Q = -L$. The set of migration rates is encoded in the weighted adjacency matrix M, which is given by $M = Q - \operatorname{diag} Q$.

The coalescence rate per deme is represented by the vector $\gamma = (\gamma_1, \gamma_2, ..., \gamma_d)$, where γ_i is the coalescence rate of deme i. Rather than fit the coalescent rates per deme as fully independent parameters, we take an approach that has the potential to favor coalescent rates that are consistent with the stationary distribution implied by the migration process (as one might expect when a system is truly at equilibrium). Let $\pi = (\pi_1, \pi_2, ..., \pi_d)^T$ denote the stationary distribution of the of the backward migration process. We then model the coalescence rate in deme i as:

$$\gamma_i = \frac{c}{\pi_i^\alpha}, \tag{S1}$$

where c is a constant scaling parameter, and $\alpha \in [0, 1]$ is a parameter that controls the relationship between coalescence rates and migration dynamics. This formulation captures a continuum of scenarios—from deme sizes being entirely independent of migration rates and constant ($\alpha = 0$) to being fully determined by the stationary distribution ($\alpha = 1$).

In this framework, the parameters (M, c, α) can be used to compute the matrix of expected pairwise coalescent times (T) and in turn the negative log likelihood l. This can be conceptualized as moving through the sequence $(M, c, \alpha) \to (M, \gamma) \to T \to l$. Here T is the matrix where the $(i, j)_{\text{th}}$ entry is the expected pairwise coalescence time between haploid samples from deme i and j, and l denotes the negative log likelihood (up to a constant). Computing T consistent with a given M and γ involves solving the equation (Strobeck, 1987)

$$\operatorname{diag}\{\gamma\} \operatorname{diag}\{T\} + LT + TL^T = 1_{d\times d}, \tag{S2}$$

where $1_{d\times d}$ is a matrix of ones. Section 1.4 will provide a detailed explanation of the solution methods for this equation.

The final step involves formulating the negative log likelihood. Since our approach employs a penalized maximum likelihood method, the objective function is expressed as $l(M, c, \alpha) + \lambda\Psi(M)$, where $\lambda\Psi(M)$ serves as a penalty term to regulate overfitting using M. The optimization task is to minimize this objective over an appropriate domain D. The explicit form of l, $\lambda\Psi$ and D, along with the rationale behind this construction are discussed in section 1.3. Section 1.2 examines the covariance structure of allele frequencies, providing essential groundwork for understanding section 1.3.

To solve the optimization problem, we utilize the L-BFGS algorithm (Byrd et al., 1995). A crucial component of this method is gradient computation, which presents notable challenges in our context. Section 1.5 elaborates on the procedures for calculating the gradient efficiently.

To select the optimal hyperparameter λ, we employ cross-validation based on the model's predictive performance in estimating allele frequencies. The details of this cross-validation procedure are provided in the Methods section of the main text; for completeness, we restate it in section 1.6.

1.2 Covariance matrix of allele frequencies

Assume we have o observed sample groups with corresponding $o \times d$ assignment matrix J if sample group i is in deme d_i, $J_{id_i} = 1$, otherwise $J_{id_i} = 0$. Assume in sample group i , we have n_i copies of haploid data on p SNPs (every diploid counts as two copies). Let allele state of sample group i , locus j , copy k be Z_{ijk} (coded as 0 or 1). As in McVean (2009), we condition on the event S that the SNP segregates in the sample due to a single mutation and take the limit as the mutation rate θ approaches zero. The expected allele state is then

$$E(Z_{ijk}|S) = \frac{\Pr(Z_{ijk}=1,S)}{P(S)} = \lim_{\theta\to 0}\frac{E(\theta t_{\mathrm{mrca}}e^{-\theta t_{\mathrm{tot}}})}{E(\theta t_{\mathrm{tot}}e^{-\theta t_{\mathrm{tot}}})} = \frac{T_{\mathrm{mrca}}}{T_{\mathrm{tot}}}, \tag{S3}$$

where the t's are random coalescence times and the T's are their expectations, under the coalescent process, or be more specific, T_{mrca} and T_{tot} are the expected height and expected total branch lengthof the coalescent tree across all samples.

Similarly, the probability that two randomly sampled (with repetition) alleles share a derived mutation is

$$E(Z_{ijk}^2|S) = \frac{T_{\mathrm{mrca}}}{T_{\mathrm{tot}}}, \tag{S4a}$$

$$E(Z_{ijk_1}Z_{ijk_2}|S) = \frac{T_{\mathrm{mrca}} - T_{d_i d_i}}{T_{\mathrm{tot}}}, \quad k_1 \neq k_2, \tag{S4b}$$

$$E(Z_{i_1jk_1}Z_{i_2jk_2}|S) = \frac{T_{\mathrm{mrca}} - T_{d_{i_1} d_{i_2}}}{T_{\mathrm{tot}}}, \quad i_1 \neq i_2. \tag{S4c}$$

Combining equation (S3) and (S4), the covariance structure of allele states is then

$$\mathrm{Var}(Z_{ijk}^2|S) = \frac{T_{\mathrm{mrca}}}{T_{\mathrm{tot}}}(1 - \frac{T_{\mathrm{mrca}}}{T_{\mathrm{tot}}}), \tag{S5a}$$

$$\mathrm{Cov}(Z_{ijk_1}, Z_{ijk_2}|S) = \frac{T_{\mathrm{mrca}}}{T_{\mathrm{tot}}}(1 - \frac{T_{\mathrm{mrca}}}{T_{\mathrm{tot}}}) - \frac{T_{d_i d_i}}{T_{\mathrm{tot}}}, \quad k_1 \neq k_2, \tag{S5b}$$

$$\mathrm{Cov}(Z_{i_1jk_1}, Z_{i_2jk_2}|S) = \frac{T_{\mathrm{mrca}}}{T_{\mathrm{tot}}}(1 - \frac{T_{\mathrm{mrca}}}{T_{\mathrm{tot}}}) - \frac{T_{d_{i_1} d_{i_2}}}{T_{\mathrm{tot}}}, \quad i_1 \neq i_2. \tag{S5c}$$

Now let $f_{ij} = \frac{\sum_{k=1}^{n_i} Z_{ijk}}{n_i}$ be the allele frequency for sample group i, locus j. We can define vectors $f_j = (f_{1j}, ..., f_{oj})^T, j = 1, 2, \ldots, p$, which can be viewed as independent realizations of the random vector of allele frequencies assuming perfect segregation. Let K be a $o \times 1$ vector such that its i_{th} entry is $\frac{1}{n_i}$, from equation (S5) and definition of f_j, the covariance matrix for each f_j can be written as

$$\Sigma = \sigma^2(1_{o\times o} - \rho \underline{T}), \tag{S6}$$

where

$$\sigma^2 = \frac{T_{\mathrm{mrca}}}{T_{\mathrm{tot}}}(1 - \frac{T_{\mathrm{mrca}}}{T_{\mathrm{tot}}}), \tag{S7a}$$

$$\rho = \frac{1}{T_{\mathrm{mrca}}(1 - \frac{T_{\mathrm{mrca}}}{T_{\mathrm{tot}}})}, \tag{S7b}$$

$$\underline{T} = JTJ^T - \mathrm{diag}\{JTJ^T\}\,\mathrm{diag}\{K\}. \tag{S7c}$$

1.3 The penalized likelihood function

To remove the mean and focus on the covariance structure, we apply a contrast matrix $C \in \mathbb{R}^{o-1\times o}$, where C is a matrix of rank $o-1$ whose rows sum to 0 . In practice, we construct C by choosing one column and setting it to -1. In each row, we then select a unique column, distinct from the chosen column, and set the corresponding entry to 1.

Assume each f_j obeys a multivariate normal distribution, the transformed data is distributed as

$$Cf_j \sim N(0, C\Sigma C^T). \tag{S8}$$

The sample covariance matrix $\widehat{\Sigma}$ after the transformation then obeys a Wishart distribution

$$C\widehat{\Sigma}C^T \sim W_{o-1}(\frac{C\Sigma C^T}{k_p}, k_p), \tag{S9}$$

where k_p represents the degrees of freedom and equals p (the number of SNPs) under perfect independence of loci (see EEMS, Petkova et al., 2016).

The negative log likelihood can then be expressed as

$$\begin{aligned} l_{\text{raw}} = & \frac{k_p}{2}\{\text{tr}[(C\Sigma C^T)^{-1}C_{ob}\widehat{\Sigma}C^T] + \log|(C\Sigma C^T)| - \log|C\widehat{\Sigma}C^T|\} \\ & + \log[\Gamma_{o-1}(\frac{k_p}{2})] - \frac{k_p(o-1)}{2}\log\frac{k_p}{2} + \frac{o}{2}\log|C\widehat{\Sigma}C^T|. \end{aligned} \tag{S10}$$

Let $\Omega = \{(i,j)|m_{ij} > 0, i = 1, \dots, d, j = 1, \dots, d\}$ denote the edge set, and let $\Omega_i = \{(i,t) \in \Omega\} \cup \{(s,i) \in \Omega\}$ denote the edges connected to node i. The penalty term is then written as $\lambda\Psi$, where

$$\Psi = \frac{1}{2}\sum_{i=1}^{d} \sum_{\substack{(i_1,j_1)\in\Omega_i, \\ (i_2,j_2)\in\Omega_i}} \frac{1}{|\Omega_i|^2}[(\frac{m_{i_1j_1}}{\widetilde{m}} - \frac{m_{i_2j_2}}{\widetilde{m}})^2 + (\frac{\widetilde{m}}{m_{i_1j_1}} - \frac{\widetilde{m}}{m_{i_2j_2}})^2], \tag{S11}$$

and $\widetilde{m}$ is the geometric mean of all the nonzero migration rates across the whole graph. The summand penalizes large differences between edge weights $m_{i_1j_1}$ and $m_{i_2j_2}$ that share a common node.

The migration rates matrix $\widehat{M}$ is estimated by solving the following penalized likelihood optimization problem:

$$\widehat{M}, \widehat{c}, \widehat{\alpha}, \widehat{k_p} = \underset{(M,c,\alpha,k_p)\in\mathcal{D}_{\text{raw}}}{\text{argmin}} (l_{\text{raw}} + \lambda\Psi) \tag{S12}$$

where $\mathcal{D}_{\text{raw}} = \mathcal{D}_{\mathcal{M}} \times R^+ \times [0,1] \times (0,p]$, $\mathcal{D}_{\mathcal{M}} = \{M|m_{ij} > 0 \text{ if } (i,j) \in \Omega,\ m_{ij} = 0 \text{ if } (i,j) \notin \Omega\}$.

Directly solving this raw optimization problem is difficult and we do several simplifications.

The first simplification involves replacing k_p with p in the negative log-likelihood function, even when there is no perfect independence of loci. Consider the function

$$F(k_p) = \log[\Gamma_{o-1}(\frac{k_p}{2})] - \frac{k_p(o-1)}{2}\log\frac{k_p}{2}, \tag{S13}$$

we would like to show that

$$\lim_{k_p\to\infty} F(k_p) - F(k_p - 1) = \frac{1-o}{2} \tag{S14}$$

Using the definition of multivariate gamma function that

$$\Gamma_a(x) = \pi^{\frac{a(a-1)}{4}} \prod_{i=1}^{a} \Gamma(x + \frac{1-i}{2}), \tag{S15}$$

the difference of the first term in $F(k_p)$ is

$$\log[\Gamma_{o-1}(\frac{k_p}{2})] - \log[\Gamma_{o-1}(\frac{k_p-1}{2})] = \log\frac{\Gamma_{o-1}(\frac{k_p}{2})}{\Gamma_{o-1}(\frac{k_p-1}{2})} = \log\frac{\Gamma(\frac{k_p}{2})}{\Gamma(\frac{k_p-o+1}{2})}. \tag{S16}$$

For the second term, we have

$$k_p \log\frac{k_p}{2} - (k_p-1)\log\frac{(k_p-1)}{2} = \log\frac{k_p}{2} + \log(1+\frac{1}{k_p-1})^{k_p-1}. \tag{S17}$$

Combining the above two equations and applying Stirling's formula yields

$$\begin{aligned} &\lim_{k_p\to\infty} F(k_p) - F(k_p-1) \\ &= \lim_{k_p\to\infty} \log\frac{\Gamma(\frac{k_p}{2})}{\Gamma(\frac{k_p-o+1}{2})(\frac{k_p}{2})^{\frac{o-1}{2}}(1+\frac{1}{k_p-1})^{\frac{(o-1)(k_p-1)}{2}}} \\ &= \frac{1-o}{2} \end{aligned} \tag{S18}$$

This means for large k_p the negative log likelihood function (neglecting constants) approximates

$$\frac{k_p}{2}[-\operatorname{tr}[(C\underline{T}C^T)^{-1}C\widehat{\Sigma}C^T] + \log|-(C\underline{T}C^T)| - \log|C\widehat{\Sigma}C^T| - \frac{o-1}{2}] \tag{S19}$$

This asymptotic linearity means that for large k_p, the optimization problem is largely independent of k_p.Therefore we can replace k_p with p and and the resulting constant can be absorbed into the penalty parameter λ. The updated optimization problem becomes

$$\widehat{M}, \widehat{c}, \widehat{\alpha} = \underset{(M,c,\alpha)\in\mathcal{D}}{\operatorname{argmin}}\ (l_p + \lambda\Psi) \tag{S20}$$

where $\mathcal{D} = \mathcal{D}_{\mathcal{M}} \times R^+ \times [0,1]$ and

$$l_p = \frac{p}{2}\{\operatorname{tr}[(C\Sigma C^T)^{-1}C_{ob}\widehat{\Sigma}C^T] + \log|(C\Sigma C^T)| - \log|C\widehat{\Sigma}C^T|\} \tag{S21}$$

The second simplification involves handling the term $C\Sigma C^T = -\frac{1}{T_{\text{tot}}}C\underline{T}C^T$, where T_{tot} is the expected total coalescence time. T_{tot} is a complicated function of the underlying model and the samples and is in general very difficult to compute. To address this, we absorb it into the model by redefining $L' = T_{\text{tot}}L$; $\gamma' = T_{\text{tot}}\gamma$, and $T' = \frac{T}{T_{\text{tot}}}$. This adjustment is justified within the maximum likelihood framework. To see this, we replace T_{tot} with its MLE given other parameters. The derivative of $l_p$with respect to T_{tot} is $\frac{p}{2}\{\operatorname{tr}[(-C\underline{T}C^T)^{-1}C\widehat{\Sigma}C^T] - \frac{o-1}{T_{\text{tot}}}\}$. Setting it to be 0, we obtain

$$T_{\text{tot}}^{\text{MLE}} = \frac{o-1}{\operatorname{tr}[(-C\underline{T}C^T)^{-1}C\widehat{\Sigma}C^T]}. \tag{S22}$$

Replacing T_{tot} in equation (S21) with this expression, the negative log likelihood function (neglecting constants) becomes

$$l_{\text{MLE}} = \frac{p}{2}\{(o-1)\log(\operatorname{tr}[(-C\underline{T}C^T)^{-1}C\widehat{\Sigma}C^T]) + \log|-C\underline{T}C^T|\} \tag{S23}$$

Notice that both l_{MLE} and $\lambda\Psi$ are invariant under the scaling of (M,c) since they are both dimensionless quantity. That means for any (M,c,α), we can define scaled parameters $M' = T_{\text{total}}^{\text{MLE}}M$, $c' = T_{\text{total}}^{\text{MLE}}c$. The new parameters (M',c',α) preserve the objective function value and satisfy $T_{\text{total}}^{\text{'MLE}} = 1$. Therefore, we can first do the optimization over $\mathcal{D}_{\text{MLE}} = \{(M,c,\alpha)|T_{\text{total}}^{\text{MLE}} = 1\}$, yielding $(\widehat{M}_{\text{MLE}}, \widehat{c}_{\text{MLE}})$, and write the solution set of minimizing $l_{\text{MLE}} + \lambda\Psi$ as $\{(x\widehat{M}_{\text{MLE}}, x\widehat{c}_{\text{MLE}}, \alpha), x \in R^+\}$. The optimization problem over $\mathcal{D}_{\text{MLE}}$ can be interpreted as finding a representative element within the solution set. By applying the constraint

$\frac{o-1}{\mathrm{tr}[(-C\underline{T}C^T)^{-1}C\widehat{\Sigma}} = 1$ and negelecting the constants, this problem becomes minimizing $\frac{p}{2}(\log|-C\underline{T}C^T|) + \lambda\Psi$ over $\bar{D}_{\mathrm{MLE}}$.

On the other hand, the negative log likelihood after T_{tot} is absorbed can be written as

$$l = \frac{p}{2}\{-\mathrm{tr}[(C\underline{T}C^T)^{-1}C\widehat{\Sigma}C^T] + \log|-C\underline{T}C^T|\} \tag{S24}$$

The optimization problem is then minimizing $l + \lambda\Psi$ over $\mathcal{D}$. For any (M, c, α), the parameters can be rescaled as $(x_0 M_0, x_0 c_0, \alpha)$, where $x_0 \in R^+$ and $(M_0, c_0, \alpha) \in \mathcal{D}_{\mathrm{MLE}}$. The optimization problem is thus equivalent to first minimizing the function over x_0 given (M_0, c_0, α), and then minimizing over all possible possible parameters in $\mathcal{D}_{\mathrm{MLE}}$. By taking the derivative of the objective function with respect to x_0 and setting it to zero, we obtain $x_0 = 1$. The solution of minimizing $l + \lambda\Psi$ over $\mathcal{D}$ thus lies in $\mathcal{D}_{\mathrm{MLE}}$, and the problem is equivalent to minimize $l + \lambda\Psi$ over D_{MLE}. By applying the constraint $\frac{o-1}{\mathrm{tr}[(-C\underline{T}C^T)^{-1}C\widehat{\Sigma}} = 1$ and negelecting the constants, the problem also reduces to minimizing $\frac{p}{2}(\log|-C\underline{T}C^T|) + \lambda\Psi$ over $\mathcal{D}_{\mathrm{MLE}}$. This is equivalent to selecting a representative of the solution set obtained after the MLE adjustment.

After these two simplifications, the final penalized likelihood function is $l + \lambda\Psi$ and the optimization problem becomes

$$\widehat{M}, \widehat{c}, \widehat{\alpha} = \underset{(M,c,\alpha)\in\mathcal{D}}{\mathrm{argmin}}\ (l + \lambda\Psi) \tag{S25}$$

1.4 Computing of the expected pairwise coalescence time

Here we would like to compute $T|M, \gamma$ from equation (S2) . We develop an algorithm to solve the more general equation

$$\operatorname{diag}\{\gamma\} \operatorname{diag}\{X\} + LX + XL^T = G. \tag{S26}$$

with time complexity $O(d^4)$, where G is an arbitrary symmetric matrix. We now introduce a few notations and give the outline of the method.

Notation	Meaning
$\otimes$	Kronecker Product
$\dagger$	Moore-Penrose inverse
$\|\|\|_F$	Frobenius Norm
vec	Vectorization
$(e_1, \ldots, e_d)$	Standard basis of R^d
$E_i, i = 1, 2, \ldots, d$	$e_i e_i^T$
g	$\text{vec}(G)$
$\epsilon_i, i = 1, 2, \ldots, d$	$\text{vec}(E_i)$
S^d	The space of $d \times d$ symmetric matrix
$X_A^0, \forall A \in S^d$	Least square solution of the equation: $LX + XL^T = A$
$X_A^1, \forall A \in S^d$	Solution of the equation $\gamma_1 E_1 \operatorname{diag}\{X\} + LX + XL^T = A$
$X_A^d, \forall A \in S^d$	Solution of the equation $\operatorname{diag}\{\gamma\} \operatorname{diag}\{X\} + LX + XL^T = A$
$\chi_A^0, \forall A \in S^d$	$\text{vec}(X_A^0)$
$\chi_A^1, \forall A \in S^d$	$\text{vec}(X_A^1)$
$\chi_A^d, \forall A \in S^d$	$\text{vec}(X_A^d)$
π	Stationary distribution, a row vector
$\hat{\pi}$	Normalized stationary distribution, i.e. $\frac{\pi}{\sqrt{\pi\pi^T}}$
ψ	$\pi \otimes \pi$
$\hat{\psi}$	$\hat{\pi} \otimes \hat{\pi}$
z	$1_{d\times 1}$
Z	zz^T
ζ	$z \otimes z$

Table S1: Notations

The remaining notations, as well as further explanations, will be provided in context. With the above notation, we proceed with the following steps:

1. Compute X_G^0 and $X_{E_i}^0$, for $i = 1, 2, \ldots, d$;
2. Compute X_G^1 and $X_{E_i}^1$, for $i = 1, 2, \ldots, d$;
3. Compute X_G^d

Before presenting the details of the three steps, we state two corollaries.

Corollary 1: The multiplicity of eigenvalue 0 is 1 for $L \otimes I + I \otimes L$, with the corresponding right eigenvector ζ and left eigenvector ψ.

Proof: In our cases, L is the Laplacian of a strongly connected graph, thus the multiplicity for eigenvalue 0 is 1, and all other eigenvalues have positive real part (Caughman and Veerman, 2006). Noting that $L = -Q$, where Q is the transition-rate matrix, we know $z = 1_{d\times 1}$ is a right eigenvector associated with eigenvalue 0.

By property of Kronecker product (Chapter 13, Laub, 2005), the eigenvalues of the matrix $L \otimes I + I \otimes L$ are of the form $\lambda_i + \lambda_j$, where λ_i and λ_j are eigenvalues of L. Since L has eigenvalue 0 with multiplicity 1, and all other eigenvalues have positive real part, the matrix $L \otimes I + I \otimes L$ must also have eigenvalue 0 with multiplicity 1; the right-eigenvector corresponding to eigenvalue 0 is $z \otimes z = 1_{d^2\times 1} = \zeta$, and the corresponding left-eigenvector is $\pi \otimes \pi = \psi$.

Corollary 2: $\gamma_1 E_1 \operatorname{diag}(X) + LX + XL^T = A$ has a unique solution for any symmetric matrix A.

Proof: Rewriting the equation in vectorized form gives

$$(\gamma_1 \epsilon_1 \epsilon_1^T + L \otimes I + I \otimes L)\operatorname{vec}(X) = \operatorname{vec}(A). \tag{S27}$$

Thus, it suffices to show that the matrix $\gamma_1 \epsilon_1 \epsilon_1^T + L \otimes I + I \otimes L$ is non-singular. Since $L \otimes I + I \otimes L$ has rank $d^2 - 1$, we only need to verify that ϵ_1 is not in the column space of $L \otimes I + I \otimes L$. If it were, it would be orthogonal to the left eigenvector $\pi \otimes \pi$. However, this is impossible because π, being the stationary distribution, has all positive entries.

Step 1

We aim to compute X_G^0 and $X_{E_i}^0$, $i = 1, 2, ..., d$, it suffices to provide an algorithm that solves the equation

$$LX + XL^T - A = 0 \tag{S28}$$

for an arbitrary symmetric matrix A.

To solve this problem, we note that getting a least square solution of equation (S28) is equivalent to solving

$$LX + XL^T = A_{\|} \tag{S29}$$

exactly, where $A_{\|}$ is the projection of A onto the range of the operator $L() + ()L^T$. This is equivalent to projecting $\operatorname{vec}(A)$ onto the range of $L \otimes I + I \otimes L$. Let V denote the range of the operator $L \otimes I + I \otimes L$, and let $V^{\perp}$ be its orthogonal complement. The projection of $\operatorname{vec}(A)$ onto V can then be written as $\operatorname{vec}(A)$ minus its projection onto $V^{\perp}$. As for $V^{\perp}$, it is simply the span of ψ^T, since

$$\begin{aligned}
&x \in V^{\perp}\\
\Leftrightarrow&\langle x, (L \otimes I + I \otimes L)y\rangle = 0, \forall y \in R^{d^2}\\
\Leftrightarrow&x^T(L \otimes I + I \otimes L)y = 0, \forall y \in R^{d^2}\\
\Leftrightarrow&x^T(L \otimes I + I \otimes L) = 0\\
\Leftrightarrow&\exists k \in R, x^T = k\psi^T \quad \text{(Corollary 1)}\\
\Leftrightarrow&x \in \operatorname{Span}(\psi^T).
\end{aligned} \tag{S30}$$

Thus, we can write

$$\operatorname{vec}(A_{\|}) = \operatorname{vec}(A) - \hat{\psi}\operatorname{vec}(A)\hat{\psi}^T, \tag{S31}$$

or in the matrix form

$$A_{\|} = A - \hat{\psi}\operatorname{vec}(A)\hat{\pi}^T\hat{\pi}. \tag{S32}$$

Now, to solve the equation $LX + XL^T = A_{\|}$ exactly, we perform a transformation. Let $P_1 = \frac{z}{\sqrt{d}}$, $P = \begin{pmatrix} P_1 & P_2 \end{pmatrix}$ be a orthonormal matrix extended from vector P_1. Define

$$L' = P^T L P, \tag{S33a}$$

$$X' = P^T X P, \tag{S33b}$$

$$A'_{\|} = P^T A_{\|} P. \tag{S33c}$$

Partition these matrices as

$$L' = \begin{pmatrix} 0 & L'_{12} \\ 0 & L'_{22} \end{pmatrix}, \tag{S34a}$$

$$X' = \begin{pmatrix} X'_{11} & X'_{12} \\ X'_{21} & X'_{22} \end{pmatrix}, \tag{S34b}$$

$$A'_{\|} = \begin{pmatrix} (A'_{\|})_{11} & (A'_{\|})_{12} \\ (A'_{\|})_{21} & (A'_{\|})_{22} \end{pmatrix}, \tag{S34c}$$

where the $()_{11}$ is the entry in row 1, column 1. From block matrix multiplication, we derive

$$X'_{22}(L'_{12})^T + L'_{22}X'_{21} = (A'_{\|})_{21}, \tag{S35a}$$

$$L'_{22}X'_{22} + X'_{22}(L'_{22})^T = (A'_{\|})_{22}. \tag{S35b}$$

Since L has eigenvalue 0 with multiplicity 1 and all other eigenvalues have positive real part, $-L$ is semi-stable. Consequently, $-L'_{22}$ is stable, ensuring that equation (S35b) has a unique solution. We solve (S35b) directly using Bartels–Stewart algorithm (Bartels and Stewart, 1972)), a standard method for solving Sylvester equations. X'_{21} can then be obtained from (S35a) . By setting $X_{11} = 0$ and using the fact that $X'_{12} = X'^T_{21}$, we fully recover X', the original solution is given by

$$X = PX'P^T. \tag{S36}$$

By choosing $A = G$ and $A = E_i$, $i = 1, 2, \ldots, d$ we can compute X^0_G and $X^0_{E_i}$.

Step 2

In this step, we compute X^1_G and $X^1_{E_i}$, for $i = 1, 2, \ldots, d$. Since X^0_G and $X^0_{E_i}$ have already been computed, it suffices to show how to compute X^1_A from X^0_A and $X^0_{E_i}$, $i = 1, 2, \ldots, d$ for arbitrary matrix A.

Let $\alpha = \mathrm{vec}(A)$, From corollary 2, equation

$$(\gamma_1\epsilon_1\epsilon_1^T + L \otimes I + I \otimes L)\chi = \alpha \tag{S37}$$

.has a unique solution

$$\chi^1_A = (\gamma_1\epsilon_1\epsilon_1^T + L \otimes I + I \otimes L)^{-1}\alpha. \tag{S38}$$

Because the Moore-Penrose pseudoinverse coincides with the inverse in this case, we can also write

$$\chi^1_A = (\gamma_1\epsilon_1\epsilon_1^T + L \otimes I + I \otimes L)^{\dagger}\alpha. \tag{S39}$$

Using the rank-one update formula for the Moore-Penrose inverse developed by Carl D. Meyer (Meyer, 1973), we can efficiently compute X^1_A . For any matrix B and vectors c_1 and c_2 where c_1 is not in the column space of B and c_2 is not in the row space of B the update is

$$(B + c_1c_2^T)^{\dagger} = B^{\dagger} - k_1u^{\dagger} - v^{\dagger}k_2^T + (1 + c_2^TB^{\dagger}c_1)v^{\dagger}u^{\dagger}, \tag{S40}$$

where

$$k_1 = B^{\dagger}c_1, \tag{S41a}$$

$$k_2^T = c_2^TB^{\dagger}, \tag{S41b}$$

$$u = (I - BB^{\dagger})c_1, \tag{S41c}$$

$$v = c_2^T(I - B^{\dagger}B). \tag{S41d}$$

The expression can be transformed to

$$\begin{aligned} &B^{\dagger} - k_1u^{\dagger} - v^{\dagger}k_2^T + (1 + c_2^TB^{\dagger}c_1)v^{\dagger}u^{\dagger} \\ &= (B^{\dagger} - k_1u^{\dagger}) + v^{\dagger}(c_2^TB^{\dagger}c_1u^{\dagger} - k_2^T) + v^{\dagger}u^{\dagger} \\ &= B^{\dagger}(I - c_1u^{\dagger}) - v^{\dagger}c_2^TB^{\dagger}(I - c_1u^{\dagger}) + v^{\dagger}u^{\dagger} \\ &= (I - v^{\dagger}c_2^T)B^{\dagger}(I - c_1u^{\dagger}) + v^{\dagger}u^{\dagger} \end{aligned} \tag{S42}$$

Since $u^\dagger$ is a vector, we have $u^\dagger = \frac{u^T}{u^T u} = \frac{c_1^T (I-BB^\dagger)^T}{c_1^T (I-BB^\dagger)^T (I-BB^\dagger) c_1}$. From the definition of Moore-Penrose inverse, we obtain

$$(I - BB^\dagger)^T = I - BB^\dagger, \tag{S43a}$$
$$BB^\dagger (I - BB^\dagger) = 0. \tag{S43b}$$

This means

$$u^\dagger = \frac{c_1^T (I - BB^\dagger)}{c_1^T (I - BB^\dagger) c_1}. \tag{S44}$$

Similarly,

$$v^\dagger = \frac{(I - B^\dagger B) c_2}{c_2^T (I - B^\dagger B) c_2}. \tag{S45}$$

Now let $B = L \otimes I + I \otimes L$, $c_1 = \gamma_1 \epsilon_1$ and $c_2 = \epsilon_1$. Since $\gamma \epsilon_1$ is not in the column space of $L \otimes I + I \otimes L$ and ϵ_1^T is not in the row space of $L \otimes I + I \otimes L$, the rank-one update formula applies. From the property of Moore-Penrose inverse, we know $B(I - B^\dagger B) = 0$, which implies $B(I - B^\dagger B) c_2 = 0$. From previous analysis, we know the kernel of $L \otimes I + I \otimes L$ is the space spanned by $\zeta = 1_{d^2 \times 1} = z \otimes z$. Therefore $(I - B^\dagger B) c_2 = k\zeta$ for some k. Replace c_2 with ϵ_1 and $(I - B^\dagger B) c_2$ with $k\zeta$, we obtain

$$v^\dagger = \frac{\zeta}{\epsilon_1^T \zeta}. \tag{S46}$$

Similarly,

$$u^\dagger = \frac{\psi^T}{\gamma_1 \psi^T \epsilon_1}. \tag{S47}$$

Substituting $v^\dagger$ and $u^\dagger$ back into the rank-one update formula gives

$$\begin{aligned} &(\gamma_1 \epsilon_1 \epsilon_1^T + L \otimes I + I \otimes L)^\dagger \\ &= (I - \frac{\zeta \epsilon_1^T}{\epsilon_1^T \zeta})(L \otimes I + I \otimes L)^\dagger (I - \frac{\epsilon_1 \psi}{\psi \epsilon_1}) + \frac{\zeta \psi}{\gamma_1 (\psi \epsilon_1)(\epsilon_1^T \zeta)}. \end{aligned} \tag{S48}$$

We can then calculate the expression of χ_A^1

$$\begin{aligned} \chi_A^1 =& (\gamma_1 \epsilon_1 \epsilon_1^T + L \otimes I + I \otimes L)^\dagger \alpha \\ =& (I - \frac{\zeta \epsilon_1^T}{\epsilon_1^T \zeta})[(L \otimes I + I \otimes L)^\dagger \alpha - (\frac{\psi \alpha}{\psi \epsilon_1})(L \otimes I + I \otimes L)^\dagger \epsilon_1] \\ &+ \frac{\psi \alpha}{\gamma_1 (\psi \epsilon_1)(\epsilon_1^T \zeta)} \zeta. \end{aligned} \tag{S49}$$

Since $(I - \frac{\zeta \epsilon_1^T}{\epsilon_1^T \zeta})\zeta = 0$, we can use any representative in $\{(L \otimes I + I \otimes L)^\dagger \alpha + k\zeta\}$ to replace $(L \otimes I + I \otimes L)^\dagger \alpha$ in the expression. From the property of Moore-Penrose pseudoinverse (Barata and Hussein, 2012) and the fact that the space spanned by ζ is the kernel of $L \otimes I + I \otimes L$, we know that finding a representative in $\{(L \otimes I + I \otimes L)^\dagger \alpha + k\zeta\}$ is equivalent to finding a symmetric matrix X such that $\mathrm{vec}(X)$ minimizes $\| (L \otimes I + I \otimes L)\mathrm{vec}(X) - \alpha \|$. This is further equivalent to finding a symmetric matrix X that minimizes $\| LX + XL^T - A \|_F$, which is simply X_A^0 solved in step 1. This means that we can replace $(L \otimes I + I \otimes L)^\dagger \alpha$ with χ_A^0 . $(L \otimes I + I \otimes L)^\dagger \epsilon_1$ can be replaced by $\chi_{E_1}^0$,with the same logic.

Using these substitutions, the expression for χ_A^1 simplifies to

$$\chi_A^1 = (I - \frac{\zeta \epsilon_1^T}{\epsilon_1^T \zeta})[\chi_A^0 - (\frac{\psi \alpha}{\psi \epsilon_1})\chi_{E_1}^0] + \frac{\psi \alpha}{\gamma_1 (\psi \epsilon_1)(\epsilon_1^T \zeta)} \zeta. \tag{S50}$$

In matrix form, this becomes

$$X_A^1 = X_A^0 - \frac{\psi \alpha}{\pi_1^2} X_{E_1}^0 + k_0 Z, \tag{S51}$$

where $k_0 = \frac{\psi^T \alpha}{\gamma_1 \pi_1^2} - X_A^0(1,1) + \frac{\psi^T \alpha}{\pi_1^2} X_{E_1}^0(1,1)$, π_i represents the i_{th} entry of y and $X_A^0(1,1)$ represents the element in row 1 column 1 of X_A^0.

Notice that we can always adjust X_A^0 in step 1 such that $X_A^0(1,1) = 0$. This can be done by substracting $X_A^0(1,1)Z$ from X_A^0. With this adjustment, the expression for X_A^1 simplifies to

$$X_A^1 = X_A^0 - \frac{\psi\alpha}{\pi_1^2} X_{E_1}^0 + \frac{\psi\alpha}{\gamma_1 \pi_1^2} Z. \tag{S52}$$

Now by letting $A = G$ and $A = E_i$, $i = 1, 2, \ldots, d$, we obtain

$$X_G^1 = X_G^0 - \frac{\psi g}{\pi_1^2} X_1^0 + \frac{\psi g}{\gamma_1 \pi_1^2} Z, \tag{S53a}$$

$$X_{E_i}^1 = X_{E_i}^0 - \frac{\pi_i^2}{\pi_1^2} X_1^0 + \frac{\pi_i^2}{\gamma_1 \pi_1^2} Z. \quad i = 1, 2, \ldots, d. \tag{S53b}$$

Step 3

In this step, we compute the final solution X_G^d .

Define the matrices

$$H_1 = \gamma_1 \epsilon_1 \epsilon_1^T + L \otimes I + I \otimes L, \tag{S54a}$$

$$H_d = \sum_{i=1}^{d} \gamma_i \epsilon_i \epsilon_i^T + L \otimes I + I \otimes L. \tag{S54b}$$

Since H_1 is non-singular, we can apply the Sherman-Morrison-Woodbury formula to effeciently compute H_d. Let $U \in R^{d^2 \times d-1}$ be a matrix where its i_{th} column is ϵ_{i+1}, and $U_\gamma \in R^{d^2 \times d-1}$be a matrix where its i_{th} column is $\gamma_i \epsilon_{i+1}$, then

$$H_d = H_1 + U U_\gamma^T. \tag{S55}$$

Applying the Sherman-Morrison-Woodbury formula, we obtain

$$H_d^{-1} = H_1^{-1} - H_1^{-1} U (I + U_\gamma^T H_1^{-1} U)^{-1} U_\gamma^T H_1^{-1}. \tag{S56}$$

We assume $I + U_\gamma^T H_1^{-1} U$ is invertible, which is valid for almost all parameter values.

Now, we compute X_G^d

$$\begin{aligned} \chi_G^d &= H_d^{-1} g \\ &= H_1^{-1} g - H_1^{-1} U (I + U_\gamma^T H_1^{-1} U)^{-1} U_\gamma^T H_1^{-1} g \\ &= \chi_G^1 - H_1^{-1} U (I + U_\gamma^T H_1^{-1} U)^{-1} U_\gamma^T \chi_G^1. \end{aligned} \tag{S57}$$

$(I + U_\gamma^T H_1^{-1} U)^{-1} U_\gamma^T \chi_G^1$ can computed by solving the equation

$$(I + U_\gamma^T H_1^{-1} U) x = U_\gamma^T \chi_G^1, \tag{S58}$$

where $U_\gamma^T \chi_G^1$ is a $d - 1 \times 1$ vector whose i_{th} entry is $\gamma_{i+1} X_G^1(i+1, i+1)$.

This equation can be written explicitly as

$$\begin{pmatrix} X_{E_2}^1(2,2) + \frac{1}{\gamma_2} & \ldots & X_{E_d}^1(d,d) \\ \vdots & \ldots & \vdots \\ X_{E_2}^1(d,d) & \ldots & X_{E_d}^1(d,d) + \frac{1}{\gamma_d} \end{pmatrix} x = \begin{pmatrix} X_G^1(2,2) \\ \vdots \\ X_G^1(d,d) \end{pmatrix}. \tag{S59}$$

After we get the solution we then compute Ux. Since i_{th} column of U is ϵ_{i+1}, this simplifies to compute

$$Ux = \sum_{i=2}^{d} x_{i-1}\epsilon_i, \tag{S60}$$

where x_i represents the i_{th} entry of x. This means

$$H_1^{-1}Ux = \sum_{i=2}^{d} x_{i-1}(H_1^{-1}\epsilon_i). \tag{S61}$$

Or in matrix form

$$X_G^d = X_G^1 - \sum_{i=2}^{d} x_{i-1}X_{E_i}^1. \tag{S62}$$

A direct method

The above method is based on rank one update of Moore-Penrose inverse and Sherman-Morrison-Woodbury formula, here we also give a direct way to find the solution. The first step is the same as the above method, i.e. computing X_G^0 and $X_{E_i}^0$, for $i = 1, 2, ..., d$. The solutions satisfy

$$LX_G^0 + X_G^0 L^T = G - \hat{\psi} g \hat{\pi}^T \hat{\pi}, \tag{S63a}$$
$$LX_{E_i}^0 + X_{E_i}^0 L^T = E_i - \hat{\pi}_i^2 \hat{\pi}^T \hat{\pi}, \quad i = 1, 2, \ldots, d. \tag{S63b}$$

The final solution is assumed to be a linear combination of X_G^0, $X_{E_i}^0$ and Z

$$X_G^d = X_G^0 - \sum_{i=1}^{d} a_i X_{E_i}^0 - bZ \tag{S64}$$

The first equation we get is the constraint that the projection cancels out

$$\hat{\psi} g - \sum_{i=1}^{d} a_i \hat{\pi}_i^2 = 0. \tag{S65}$$

Apply the operator $L() + ()L^T$ to X_G^d we get

$$LX_G^d + X_G^d L^T = G - \sum_{i=1}^{d} a_i E_i. \tag{S66}$$

Combining with equation (S26) , from the diagonal elements we obtain another d equations

$$X_G^0(j,j) - \sum_{i=1}^{d} a_i X_{E_i}^0(j,j) - b = \frac{a_j}{\gamma_j}, \quad i = 1, 2, \ldots, d. \tag{S67}$$

The $d+1$ linear equations of the $d+1$ unknowns $\{b, a_i, 1 \le i \le d\}$ can then be written in matrix notation

$$\begin{pmatrix} 0 & \hat{\pi}_1^2 & \ldots & \hat{\pi}_d^2 \\ 1 & X_{E_1}^0(1,1) + \frac{1}{\gamma_1} & \ldots & X_{E_d}^0(1,1) \\ \vdots & \vdots & \ldots & \vdots \\ 1 & X_{E_1}^0(d,d) & \ldots & X_{E_d}^0(d,d) + \frac{1}{\gamma_d} \end{pmatrix} \begin{pmatrix} b \\ a_1 \\ \vdots \\ a_d \end{pmatrix} = \begin{pmatrix} \hat{\psi} g \\ X_G^0(1,1) \\ \vdots \\ X_G^0(d,d) \end{pmatrix}. \tag{S68}$$

The solution is obtained by solving the above linear system, which provides values for b and a_i, $i = 1, 2, \ldots, d$. These coefficients then fully determine X_G^d.

Summary

The total time complexity of both algorithms is $O(d^4)$, primarily due to solving $O(d)$ Lyapunov equations in step 1. Similarly, the total space complexity is $O(d^3)$, as it is dominated by storing $O(d)$ matrices during the same step. In practice, the first method is preferred for its robustness. By selecting the deme with the largest π_i and performing a rank-1 update on it first, we effectively control the numerical computation's size, ensuring stability and efficiency. The second method, while easier to understand, can serve as an intuitive illustration.

1.5 Computing of the gradient

The gradient of the object function is the sum of the gradient of l, and $\lambda\Psi_\lambda$. In this section we show how to compute the gradient of the three components.

Computating ∇l

We start by showing how to compute $\frac{\partial l}{\partial m_{st}}$ for a given m_{st} where $(s,t) \in \Omega$. Let T be the solution of equation (S2) and let $\tau = \text{vec}(T)$. Using the chain rule, we have

$$\frac{\partial l}{\partial m_{st}} = \frac{\partial l}{\partial \tau}\frac{\partial \tau}{\partial m_{st}}, \tag{S69}$$

where $\frac{\partial l}{\partial \tau}$ is a $1 \times d^2$ row vector and $\frac{\partial \tau}{\partial L_{st}}$ is a $d^2 \times 1$ column vector.

We first compute $\frac{\partial l}{\partial \tau}$. From the expression

$$l = \frac{p}{2}\{-\operatorname{tr}[(C\underline{T}C^T)^{-1}C\widehat{\Sigma}C^T] + \log|-C\underline{T}C^T|\}, \tag{S70}$$

we know

$$\frac{\partial l}{\partial C\underline{T}C^T} = \frac{p}{2}[(C\underline{T}C^T)^{-1}C\widehat{\Sigma}C^T(C\underline{T}C^T)^{-1} + (C\underline{T}C^T)^{-1}]. \tag{S71}$$

From this and the fact that

$$\frac{\partial l}{\partial \underline{T}} = C^T\frac{\partial l}{\partial C\underline{T}C^T}C, \tag{S72}$$

we can compute $\frac{\partial l}{\partial T}$ using

$$\frac{\partial l}{\partial T} = J^T\left[\frac{\partial l}{\partial \underline{T}} - \text{diag}\left\{\frac{\partial l}{\partial \underline{T}}\right\}\text{diag}\{K\}\right]J, \tag{S73}$$

(this is because $\underline{T} = JTJ^T - \text{diag}\{JTJ^T\}\,\text{diag}\{K\}$). $\frac{\partial l}{\partial \tau}$ can then be computed from

$$\frac{\partial l}{\partial \tau} = [\text{vec}(\frac{\partial l}{\partial T})]^T. \tag{S74}$$

Next, we compute $\frac{\partial l}{\partial \tau}\frac{\partial \tau}{\partial m_{st}}, (s,t) \in \Omega$ given $\frac{\partial l}{\partial \tau}$. From the last section, we know $H_d\tau = \zeta$. This implies

$$\frac{\partial H_d}{\partial m_{st}}\tau + H_d\frac{\partial \tau}{\partial m_{st}} = 0. \tag{S75}$$

Thus,

$$H_d\frac{\partial \tau}{\partial m_{st}} = -\frac{\partial H_d}{\partial m_{st}}\tau, \tag{S76}$$

This means

$$\frac{\partial \tau}{\partial m_{st}} = -H_d^{-1}\frac{\partial H_d}{\partial m_{st}}\tau, \tag{S77}$$

and

$$\frac{\partial l}{\partial \tau}\frac{\partial \tau}{\partial m_{st}} = -(\frac{\partial l}{\partial \tau}H_d^{-1})\frac{\partial H_d}{\partial m_{st}}\tau. \tag{S78}$$

Notice that

$$\frac{\partial H_d}{\partial m_{st}} = \frac{\partial H_d}{\partial L_{ss}} - \frac{\partial H_d}{\partial L_{st}} + \sum_{i=1}^{d}\frac{\partial H_d}{\partial \gamma_i}\frac{\partial \gamma_i}{\partial m_{st}}, \tag{S79}$$

we have

$$-(\frac{\partial l}{\partial \tau}H_d^{-1})\frac{\partial H_d}{\partial m_{st}}\tau = (\frac{\partial l}{\partial \tau}H_d^{-1})(\frac{\partial H_d}{\partial L_{st}} - \frac{\partial H_d}{\partial L_{ss}})\tau - \sum_{i=1}^{d}\frac{\partial \gamma_i}{\partial m_{st}}(\frac{\partial l}{\partial \tau}H_d^{-1})\frac{\partial H_d}{\partial \gamma_i}\tau. \tag{S80}$$

The computation of the right-hand side can be divided into three parts, i.e. computing $\frac{\partial l}{\partial \tau} H_d^{-1}$, computing $(\frac{\partial H_d}{\partial L_{ss}} - \frac{\partial H_d}{\partial L_{st}})\tau$ as well as $\frac{\partial H_d}{\partial \gamma_i}\tau$, and computing $\frac{\partial \gamma_i}{\partial m_{st}}$.

For the second part, the expressions

$$(\frac{\partial H_d}{\partial L_{st}} - \frac{\partial H_d}{\partial L_{ss}})\tau = \text{vec}(e_s e_t^T T + T e_t e_s^T) - \text{vec}(e_s e_s^T T + T e_s e_s^T) \tag{S81}$$

and

$$\frac{\partial H_d}{\partial \gamma_i}\tau = \epsilon_i \epsilon_i^T \tau = (\epsilon_i^T \tau)\epsilon_i \tag{S82}$$

are easy to calculate as long as we get T.

For the third part, since $\gamma_i = \frac{c}{\pi_i^\alpha}$, we know

$$\frac{\partial \gamma_i}{\partial m_{st}} = \frac{-c}{\pi_i^{\alpha+1}} \frac{\partial \pi_i}{\partial m_{st}} = \frac{-\gamma_i}{\pi_i} \frac{\partial \pi_i}{\partial m_{st}}. \tag{S83}$$

On the other hand,

$$\frac{\partial \pi}{\partial m_{st}} = \frac{\partial \pi}{\partial L_{ss}} - \frac{\partial \pi}{\partial L_{st}}. \tag{S84}$$

Since $\pi L = 0$ and $\pi z = 1$, we have

$$\pi \frac{\partial L}{\partial L_{st}} + \frac{\partial \pi}{\partial L_{st}} L = 0, \tag{S85}$$

and

$$\frac{\partial \pi}{\partial L_{st}} z = 0. \tag{S86}$$

This means

$$\frac{\partial \pi}{\partial L_{st}}(L + Z) = -\pi \frac{\partial L}{\partial L_{st}}, \tag{S87}$$

which implies

$$\frac{\partial \pi}{\partial L_{st}} = -\pi \frac{\partial L}{\partial L_{st}}(L + Z)^{-1}, \tag{S88}$$

and

$$\frac{\partial \pi}{\partial m_{st}} = -\pi(\frac{\partial L}{\partial L_{ss}} - \frac{\partial L}{\partial L_{st}})(L + Z)^{-1}. \tag{S89}$$

We then obtain

$$\frac{\partial \pi_i}{\partial m_{st}} = -\pi(\frac{\partial L}{\partial L_{ss}} - \frac{\partial L}{\partial L_{st}})(L + Z)^{-1} e_i. \tag{S90}$$

Now we only need to compute the first part

$$\frac{\partial l}{\partial \tau} H_d^{-1} = [(H_d^T)^{-1}(\frac{\partial l}{\partial \tau})^T]^T, \tag{S91}$$

which further reduces to the computation of

$$(H_d^T)^{-1}(\frac{\partial l}{\partial \tau})^T. \tag{S92}$$

However, we know

$$H_d^T = \sum_{i=1}^{d} \gamma_i \epsilon_i \epsilon_i^T + L^T \otimes I + I \otimes L^T, \tag{S93}$$

thus we only need to solve the equation

$$\text{diag}\{\gamma\}\,\text{diag}\{X\} + L^T X + XL = \frac{\partial l}{\partial T}. \tag{S94}$$

Let T^{∇} be the solution to the equation. Then $(\frac{\partial l}{\partial \tau} H_d^{-1}) \frac{\partial H_d}{\partial L_{st}} \tau$ is the summation of entries of $T^{\nabla} \odot (e_s e_t^T T + T e_t e_s^T)$, where $\odot$ represents the Hadamard product. This is simply twice the inner product of the s_{th} column of T^{∇} and the t_{th} column of T. The expression $(\frac{\partial l}{\partial \tau} H_d^{-1})(\frac{\partial H_d}{\partial L_{st}} - \frac{\partial H_d}{\partial L_{ss}})\tau$ can be computed. Similarly, since

$$\frac{\partial H_d}{\partial \gamma_i} \tau = (\epsilon_i^T \tau) \epsilon_i, \tag{S95}$$

we have

$$(\frac{\partial l}{\partial \tau} H_d^{-1}) \frac{\partial H_d}{\partial \gamma_i} \tau = T_{ii}^{\nabla} T_{ii}, \tag{S96}$$

where T_{ii}^{∇} and T_{ii} represents the $(i,i)_{\text{th}}$ entry of T^{∇} and T. Combining this equation with equation (S83), we have

$$-\sum_{i=1}^{d} \frac{\partial \gamma_i}{\partial m_{st}} (\frac{\partial l}{\partial \tau} H_d^{-1}) \frac{\partial H_d}{\partial \gamma_i} \tau = \pi (\frac{\partial L}{\partial L_{st}} - \frac{\partial L}{\partial L_{ss}})(L+Z)^{-1} [\sum_{i=1}^{d} \frac{\gamma_i}{\pi_i} T_{ii}^{\nabla} T_{ii} e_i] \tag{S97}$$

Let x be a vector such that

$$(L+Z)x = \delta, \tag{S98}$$

where $\delta = \sum_{i=1}^{d} \frac{\gamma_i}{\pi_i} T_{ii}^{\nabla} T_{ii} e_i$ is a vector such that its i_{th} element is $\frac{\gamma_i}{\pi_i} X_{ii}^{\nabla} T_{ii}$. The expression $-\sum_{i=1}^{d} \frac{\partial \gamma_i}{\partial m_{st}} (\frac{\partial l}{\partial \tau} H_d^{-1}) \frac{\partial H_d}{\partial \gamma_i} \tau$ can then be reduced to $\pi_s (x_t - x_s)$, where x_t represents the t_{th} entry of x.

Now we only need to solve the equation (S94). The method is similar to that shown in section 1.4. The only difference is that for L^T the right-eigenvector corresponding to eigenvalue 0 is π^T, and the corresponding left eigenvector is z^T. After we correct for this difference, the procedures are exactly the same. We can compute this with time complexity $O(d^4)$ and space complexity $O(d^3)$. To be more precise, we go through the following procedure to compute the solution of the equation

$$\text{diag}\{\gamma\} \, \text{diag}\{X\} + L^T X + XL = G. \tag{S99}$$

1.Compute the exact least square solutions of

$$L^T X + XL = G, \tag{S100a}$$

$$L^T X + XL = E_i, \quad i = 1, 2, \ldots, d. \tag{S100b}$$

It suffices to solve

$$L^T X + XL = A \tag{S101}$$

for arbitrary symmetric matrix A in the least square sense.

To so this, we project A onto the range of $L^T() + ()L$. Similar to the previous analysis, we see that the projection is

$$A_{\parallel} = A - \frac{1}{d^2} \zeta^T \text{vec}(A) Z. \tag{S102}$$

We then perform the same transformation, i.e.

$$(L^T)' = P^T L^T P = (L')^T, \tag{S103a}$$

$$X' = P^T X P, \tag{S103b}$$

$$A'_{\parallel} = P^T A_{\parallel} P, \tag{S103c}$$

and use the following block division

$$L' = \begin{pmatrix} 0 & 0 \\ (L'_{12})^T & (L'_{22})^T \end{pmatrix}, \tag{S104a}$$

$$X' = \begin{pmatrix} X'_{11} & X'_{12} \\ X'_{21} & X'_{22} \end{pmatrix}, \tag{S104b}$$

$$A'_{\parallel} = \begin{pmatrix} (A'_{\parallel})_{11} & (A'_{\parallel})_{12} \\ (A'_{\parallel})_{21} & (A'_{\parallel})_{22} \end{pmatrix}. \tag{S104c}$$

The equations to be solved are then

$$(L'_{12})^T X'_{11} + (L'_{22})^T X'_{21} = (A'_{\|})_{21}, \tag{S105a}$$

$$L'_{22}X'_{22} + X'_{22}(L'_{22})^T = (A'_{\|})_{22} - (L'_{12})^T (X'_{21})^T - X'_{21}L'_{12}. \tag{S105b}$$

Setting $X_{11} = 0$, we can then get X'_{21} from the first equation. The second equation is a canonical Lyapunov equation, and we can solve X'_{22} from it. Using the fact that $X'_{12} = X'^T_{21}$, we get X', and the solution X can then be obtained from $PX'P^T$. By setting $A = G$ and $A = E_i$, $i = 1, 2, \ldots, d$, we obtain the solutions.

2. Perform a rank-1 update similar to step 2 in section 1.4, switching between L and L^T, as well as between ζ and ψ^T in $v^\dagger$ and $u^\dagger$. Define X^0_A as the least-squares solution of $L^T X + XL = A$, and χ^0_A as its vectorization. Consistent with the conventions established in section 1.4 (except for replacing L with L^T), introduce $X^1_A, \chi^1_A, X^d_A, \chi^d_A$. We obtain

$$\chi^1_G = \left(I - \frac{\psi^T \epsilon_1^T}{\psi \epsilon_1}\right)\left[\chi^0_G - \left(\frac{\zeta^T g}{\zeta^T \epsilon_1}\right)\chi^0_{E_1}\right] + \frac{\zeta^T g}{\gamma_1(\zeta^T\epsilon_1)(\psi\epsilon_1)}\psi^T, \tag{S106a}$$

$$\chi^1_{E_i} = \left(I - \frac{\psi^T \epsilon_1^T}{\psi \epsilon_1}\right)\left[\chi^0_{E_i} - \left(\frac{\zeta^T \epsilon_i}{\zeta^T \epsilon_1}\right)\chi^0_{E_1}\right] + \frac{\zeta^T \epsilon_i}{\gamma_1(\zeta^T\epsilon_1)(\psi\epsilon_1)}\psi^T, \quad i = 1, 2, \ldots, d. \tag{S106b}$$

By adjusting $X^0_{E_i}$ such that $X^0_{E_i}(1,1) = 0$ (through subtracting $\frac{X^0_{E_i}(1,1)}{\pi_1^2}\psi$ from $X^0_{E_i}$), the equation in matrix form can then be written as

$$X^1_G = X^0_G - (\zeta^T\epsilon_0)X^0_{E_1} + \frac{\zeta^T\epsilon_0}{\gamma_1\pi_1^2}\pi^T\pi, \tag{S107a}$$

$$X^1_{E_i} = X^0_{E_i} - X^0_{E_1} + \frac{1}{\gamma_1\pi_1^2}\pi^T\pi, \quad i = 1, 2, \ldots, d. \tag{S107b}$$

3. Use the Sherman-Morrison-Woodbury formula similar to step 3 in the last section, solving

$$\left[I + U_\gamma^T (H_1^{-1})^T U\right] x = U_\gamma^T \chi^1_G \tag{S108}$$

and obtaining the solution through

$$X^d_G = X^1_G - \sum_{i=2}^{d} x_{i-1} X^1_{E_i}. \tag{S109}$$

By setting $G = \frac{\partial l}{\partial T}$, we can compute the solution T^∇.

For the gradient, we also need to compute $\frac{\partial l}{\partial c}$ and $\frac{\partial l}{\partial \alpha}$. Similar to previous calculations, we know

$$\frac{\partial l}{\partial \gamma_i} = -\left(\frac{\partial l}{\partial \tau} H_d^{-1}\right)\frac{\partial H_d}{\partial \gamma_i}\tau = -T^\nabla_{ii} T_{ii}. \tag{S110}$$

Thus

$$\frac{\partial l}{\partial c} = \sum_{i=1}^{d} \frac{\partial l}{\partial \gamma_i}\frac{\partial \gamma_i}{\partial c} = \sum_{i=1}^{d} \frac{\partial l}{\partial \gamma_i}\frac{1}{\pi_i^\alpha} = -\sum_{i=1}^{d} \frac{1}{\pi_i^\alpha} T^\nabla_{ii} T_{ii}, \tag{S111a}$$

$$\frac{\partial l}{\partial \alpha} = \sum_{i=1}^{d} \frac{\partial l}{\partial \gamma_i}\frac{\partial \gamma_i}{\partial \alpha} = -\sum_{i=1}^{d} \frac{\partial l}{\partial \gamma_i}\frac{c}{\pi_i^\alpha}\log(\alpha) = \sum_{i=1}^{d} \frac{c\log(\alpha)}{\pi_i^\alpha} T^\nabla_{ii} T_{ii}. \tag{S111b}$$

The gradients of l are then fully determined.

Computing $\nabla\Psi$

The penalty function is

$$\Psi = \frac{1}{2}\sum_{i=1}^{d} \sum_{\substack{(i_1,j_1)\in\Omega_i,\\(i_2,j_2)\in\Omega_i}} \frac{1}{|\Omega_i|^2}\left[\left(\frac{m_{i_1j_1}}{\widetilde{m}} - \frac{m_{i_2j_2}}{\widetilde{m}}\right)^2 + \left(\frac{\widetilde{m}}{m_{i_1j_1}} - \frac{\widetilde{m}}{m_{i_2j_2}}\right)^2\right]. \tag{S112}$$

To compute its gradient, we first consider the gradient of the first term $\frac{1}{2}\sum_{i=1}^{d} \sum_{\substack{(i_1,j_1)\in\Omega_i,\\(i_2,j_2)\in\Omega_i}} \frac{1}{|\Omega_i|^2}\left(\frac{m_{i_1j_1}}{\widetilde{m}} - \frac{m_{i_2j_2}}{\widetilde{m}}\right)^2$.

To simplify the notation, we let $g_{st} = \frac{m_{st}}{\widetilde{m}}$. The first term then becomes $\frac{1}{2}\sum_{i=1}^{d} \sum_{\substack{(i_1,j_1)\in\Omega_i,\\(i_2,j_2)\in\Omega_i}} \frac{1}{|\Omega_i|^2}(g_{i_1j_1} - g_{i_2j_2})^2$.

Using the identity

$$\frac{1}{2}\sum_{\substack{(i_1,j_1)\in\Omega_i,\\(i_2,j_2)\in\Omega_i}} (g_{i_1j_1} - g_{i_2j_2})^2 = |\Omega_i|\left(\sum_{(s,t)\in\Omega_i} g_{st}^2\right) - \left(\sum_{(s,t)\in\Omega_i} g_{st}\right)^2, \tag{S113}$$

we find

$$\begin{aligned}&\frac{1}{2}\sum_{i=1}^{d} \sum_{\substack{(i_1,j_1)\in\Omega_i,\\(i_2,j_2)\in\Omega_i}} \frac{1}{|\Omega_i|^2}(g_{i_1j_1} - g_{i_2j_2})^2\\ &= \sum_{i=1}^{d}\left[\frac{1}{|\Omega_i|}\left(\sum_{(s,t)\in\Omega_i} g_{st}^2\right) - \frac{1}{|\Omega_i|^2}\left(\sum_{(s,t)\in\Omega_i} g_{st}\right)^2\right].\end{aligned} \tag{S114}$$

This expression, denoted as Ψ^g, represents the penalty on the variance of edge weights within nodes. The gradient with respect to m_{ij} is then given by

$$\frac{\partial\Psi^g}{\partial m_{ij}} = \sum_{(s,t)\in\Omega} \frac{\partial\Psi^g}{\partial g_{st}}\frac{\partial g_{st}}{\partial m_{ij}}, \tag{S115}$$

where

$$\frac{\partial\Psi^g}{\partial g_{st}} = 2\left[\left(\frac{1}{|\Omega_s|} + \frac{1}{|\Omega_t|}\right) g_{st} - \frac{1}{|\Omega_s|}\sum_{(i',j')\in\Omega_s} g_{i'j'} - \frac{1}{|\Omega_t|}\sum_{(i',j')\in\Omega_t} g_{i'j'}\right]. \tag{S116}$$

For $\frac{\partial g_{st}}{\partial m_{ij}}$, we use the equation

$$\log(\widetilde{m}) = \frac{1}{|\Omega|}\sum_{(i',j')\in\Omega} \log(m_{i'j'}) \tag{S117}$$

and obtain

$$\frac{\partial g_{st}}{\partial m_{ij}} = \frac{1}{\widetilde{m}}\left(I\{(i,j) = (s,t)\} - \frac{1}{|\Omega|}\frac{m_{st}}{m_{ij}}\right), \tag{S118}$$

where $I\{(i,j) = (s,t)\}$ is the indicator function. Similarly, let $h_{st} = \frac{\widetilde{m}}{m_{st}}$ and denote the second term by Ψ^h, we have

$$\frac{\partial\Psi^h}{\partial m_{ij}} = \sum_{(s,t)\in\Omega} \frac{\partial\Psi^h}{\partial h_{st}}\frac{\partial h_{st}}{\partial m_{ij}}, \tag{S119}$$

where

$$\frac{\partial\Psi^h}{\partial h_{st}} = 2\left[\left(\frac{1}{|\Omega_s|} + \frac{1}{|\Omega_t|}\right) h_{st} - \frac{1}{|\Omega_s|}\sum_{(i',j')\in\Omega_s} h_{i'j'} - \frac{1}{|\Omega_t|}\sum_{(i',j')\in\Omega_t} h_{i'j'}\right]. \tag{S120}$$

For $\frac{\partial h_{st}}{\partial m_{ij}}$, we obtain

$$\frac{\partial h_{st}}{\partial m_{ij}} = \frac{-h_{st}^2}{\widetilde{m}} \left(I\{(i,j) = (s,t)\} - \frac{1}{|\Omega|} \frac{m_{st}}{m_{ij}} \right). \tag{S121}$$

The total gradient $\frac{\partial \Psi}{\partial m_{ij}}$ is then given by

$$\frac{\partial \Psi}{\partial m_{ij}} = \frac{\partial \Psi^g}{\partial m_{ij}} + \frac{\partial \Psi^h}{\partial m_{ij}}. \tag{S122}$$

2 Settings of simulations and empirical datasets

2.1 Non-equilibrium simulations

The non-equilibrium simulations are also performed using msprime (Baumdicker et al., 2022). For the pulse migration scenario, we assume a uniform base migration rate across the graph. Forward in time, the migration process takes place at discrete time points $4i\Delta t_m$, where $i = 1, 2, \ldots$, and Δt_m represents the time interval between mass migration events. At each of these time points, the frontier populations of the migration wave—beginning with the populations in the central column and progressing outward until reaching the boundary columns—split and send individuals to adjacent demes. During each mass migration event, let population A denote the source population and population B the recipient population. After the migration, 50% of the individuals in population B will originate from population A.

Backward in time, this scenario represents populations migrating from the boundary columns towards the central column. In each mass migration event, 50% of the individuals in population B will migrate backward to population A, effectively reversing the direction of movement observed in forward time.

It's important to mention that the uniform base migration happens parallel to mass migration events, and will effectively dilute the effect of the mass migration events. This is different from the case of range expansion, as we shall see.

Forward in time, the range expansion process occurs at discrete time points $4i\Delta t_r$, where $n = 1, 2, \ldots$, and Δt_r represents the time interval between range expansion events. At each of these time points, the frontier populations of the migration wave—starting from the populations in the central column and spreading outward until reaching the boundary columns—split and send 10% of their individuals to occupy the adjacent vacant demes. Initially, all demes, except for those in the central column, are vacant. The newly occupied demes then undergo a period of exponential growth from $4i\Delta t_r$ to $(4i+1)\Delta t_r$, during which their population size reaches that of their ancestral population. These demes remain at this size until the next range expansion event at $4(i+1)\Delta t_r$.

Backward in time, this process corresponds to the newly occupied demes undergoing exponential shrinkage and merging with their ancestral deme, such that the merged population consists of 10% lineages from the newly occupied deme and 90% lineages from the ancestral deme.

Parameters of our range expansion model are adapted from the human Out-of-Africa model (Ramachandran et al., 2005), where Δt_r is very small due to the rapid expansion. However, because our model includes only a small number of demes, the total duration is short—approximately $20\Delta t_r$—and the effect of base migration would be minor, even when included. This differs from the original Out-of-Africa model, which involves over 200 demes, making careful model comparisons that account for base migration necessary.

2.2 Empirical datasets

The gray wolf dataset originates from Schweizer et al. (2016). It was initially used in FEEMS (Marcus et al., 2021) and later corrected by (cite Viv). In our research, we utilize the corrected version.

The poplars (*Populus trichocarpa/balsamifera*) dataset originates from Geraldes et al. (2014) and was later adapted by Lundgren and Ralph (2019) for their analysis. In our research, we used this adapted version.

The white-crowned manakin dataset comes from (Berv et al., 2021), and we directly use the data employed by the researchers in their EEMS analysis.

All ancient human datasets were derived from the 1240k dataset provided by the Allen Ancient DNA Resource (Mallick et al., 2024; Mallick and Reich, 2023), with a focus on autosomal data. Initially, we filtered the dataset based on temporal and geographic criteria, including time period, longitude, and latitude.

For the $7000-3500$ BCE and $4500-3500$ BCE datasets, we included all individuals from the respective periods within the Europe block (longitude $\in [-10°, 35°]$, latitude $\in [32°, 55°]$) and the Eurasia block (longitude $\in [35°, 55°]$, latitude $\in [25°, 55°]$).

For the $3500-1500$ BCE and $2500-1500$ BCE dataset, we selected all individuals from the respective periods within the Europe sample block (lon $\in [-10°, 35°]$, lat $\in [32°, 55°]$) and the Extended Eurasia sample block (lon $\in [35°, 75°]$, lat $\in [25°, 55°]$).

For all four datasets, we then applied the following filters using plink(Purcell et al., 2007):

Filter (plink name)	Parameters
Minor allele frequency (maf)	0.001
SNP missingness (geno)	0.4
Individual missingness (mind)	0.4
Minor allele frequency (maf)	0.01
Linkage disequilibrium (indep-pairwise)	50, 5, 0.2

Table S2: Filters

The first minor allele frequency filter was applied to remove loci with no variation in the smaller dataset. The SNP missingness filter and the individual missingness filter were used to increase the genotype rate. The second minor allele frequency filter was applied to remove newly occurring mutations that have not been fully spread and thus do not contain sufficient geographical information. The linkage disequilibrium filter was applied to retain SNPs that exhibit nearly perfect segregation. After filtering, all datasets achieved a genotype rate around 90%.

The final step after the filtering procedures involved removing duplicate samples from the dataset, as some samples had been sequenced multiple times using different methods and sequencing depths. References for the samples used in each dataset are provided below. And lists of samples used in each dataset are available at https://github.com/ShenHaotv/frame-analysis.

Study(Abbreviation in AADR)	Samples	Reference
AllentoftNature2024	17	(3)
AntonioGaoMootsScience2019	4	(5)
BraceDiekmannNatureEcologyEvolution2019	9	(12)
CassidyNature2020	18	(16)
ChildebayevaHaakMolBioEvo2022	24	(18)
FeldmanNatureCommunications2019	1	(21)
FernandesNatureEcologyEvolution2020	7	(23)
FernandesScientificReports2018	11	(22)
FowlerOlaldeNature2021	25	(24)
FreilichPinhasiScientificReports2021	15	(25)
FurtwanglerNatureCommunications2020	5	(26)
GambaNatureCommunications2014	4	(27)
GelabertBioRxiv2023	170	(29)
GelabertSciRep2022	9	(28)
GokhmanNatureCommunications2020	1	(31)
GonzalesFortesCurrentBiology2017	4	(32)
HarneyCheronetGenomeResearch2021	4	(36)
HarneyMayNatureCommunications2018	13	(35)
HofmanovaPNAS2016	5	(37)
JensenSchroederNatureCommunications2019	1	(41)
LazaridisAlpaslanRoodenbergScience2022	62	(49)
LazaridisNature2014	3	(46)
LazaridisNature2016	12	(47)
LazaridisNature2017	1	(48)
LipsonNature2017	42	(51)
MarchiExcoffierCell2022	13	(57)
MarcusNatureCommunications2020	3	(59)
MarotiTorokCurrBio2022	1	(61)
MartinianoPLoSGenetics2017	4	(60)
MathiesonNature2015	35	(62)
MathiesonNature2018	79	(63)
MattilaCommBio2023	5	(64)
NarasimhanPattersonScience2019	3	(69)
NovakPLoSOne2021	29	(70)
OlaldeMBE2015	1	(71)
OlaldeNature2018	15	(72)
OlaldeScience2019	4	(73)
PapacScienceAdvances2021	26	(74)
PattersonNature2021	15	(75)
PenskeHaakNature2023	79	(76)
PosthYuNature2023	13	(79)
RivollatNature2023	64	(83)
RivollatScienceAdvance2020	64	(82)
RohlandMallickGenomeResearch2022	3	(84)
SanchezQuintoPNAS2019	3	(95)
ScheibAnnHumBio2019	1	(86)
SimoesPNAS2024	7	(91)
SimoesNature2023	5	(90)
SkourtaniotiCell2020	16	(92)
VillalbaMoucoCurrentBiology2019	4	(97)
WangNatureCommunications2019	7	(99)
YuvandeLoosdrechtiScience2022	9	(101)

Table S3: References for samples used in the 7000-3500 BCE dataset

Study (Abbreviation in AADR)	Samples	Reference
AgranatTamirWaldmanCell2020	17	(1)
AllentoftNature2015	7	(2)
AllentoftNature2024	3	(3)
AmorimNatureCommunications2018	1	(4)
AntonioGaoMootsScience2019	3	(5)
ArianoCell2022	3	(6)
BlocherPNAS2023	32	(11)
BraceDiekmannNatureEcologyEvolution2019	4	(12)
BrunelPNAS2020	1	(13)
CassidyNature2020	19	(16)
CassidyPNAS2016	1	(15)
ClementeCell2021	6	(19)
DamgaardScience2018	9	(20)
FernandesNatureEcologyEvolution2020	19	(23)
FernandesScientificReports2018	6	(22)
FowlerOlaldeNature2021	1	(24)
FreilichPinhasiScientificReports2021	7	(25)
FurtwanglerNatureCommunications2020	69	(26)
GambaNatureCommunications2014	1	(27)
GelabertSciRep2022	1	(28)
GonzalesFortesCurrentBiology2017	1	(32)
GonzalesFortesProcRoyalSocB2019	3	(33)
HaberAJHG2017	4	(34)
HarneyCheronetGenomeResearch2021	1	(36)
ImmelCommBiol2021	33	(39)
ImmelNature2020	2	(38)
IngmanStockhammerPLoS2021	8	(40)
JeongNatureEcologyEvolution2019	1	(42)
KoptekinCurrentBiology2023	7	(43)
KrzewinskaScienceAdvances2018	6	(44)
LazaridisAlpaslanRoodenbergScience2022	90	(49)
LazaridisNature2016	6	(47)
LazaridisNature2017	4	(48)
LinderholmNatureScientificReports2020	4	(50)
LipsonNature2017	16	(51)
MaierFlegontovELife2023	1	(53)
MalmstromProcBiolSci2019	1	(56)
MarcusNatureCommunications2020	21	(59)
MartinianoPLoSGenetics2017	6	(60)
MathiesonNature2015	37	(62)
MathiesonNature2018	23	(63)
MittnikScience2019	20	(67)
MootsNatEcolEvol2023	2	(68)
NarasimhanPattersonScience2019	130	(69)
OlaldeNature2018	157	(72)
OlaldeScience2019	59	(73)
PapacScienceAdvances2021	140	(74)
PattersonNature2021	74	(75)
PenskeHaakNature2023	12	(76)
PenskeSciRep2024	40	(77)
PosthYuNature2023	2	(79)
RivollatScienceAdvance2020	1	(82)
RohlandMallickGenomeResearch2022	4	(84)
SaupeScheibCurrBio2021	1	(85)
ScheibAnnHumBio2019	1	(86)
SchroederPNAS2019	17	(87)
SeguinOrlandoCurrBio2021	9	(89)
SkourtaniotiCell2020	41	(92)
SkourtaniotiRingbauerNatureEcologyEvolution2023	28	(93)
ValdioseraPNAS2018	3	(96)
VillalbaMoucoSciAdv2021	88	(98)
WangKrauseCellGenomics2023	1	(100)
WangNatureCommunications2019	28	(99)

Continued on next page

Study (Abbreviation in AADR)	Samples	Reference
ZegaracScientificReports2021	18	(102)

Table S4: References for samples used in the 3500-1500 BCE dataset

Study (Abbreviation in AADR)	Samples	Reference
AllentoftNature2024	3	(3)
BraceDiekmannNatureEcologyEvolution2019	15	(12)
CassidyNature2020	17	(16)
FernandesNatureEcologyEvolution2020	3	(23)
FernandesScientificReports2018	10	(22)
FowlerOlaldeNature2021	27	(24)
FreilichPinhasiScientificReports2021	13	(25)
FurtwanglerNatureCommunications2020	2	(26)
GambaNatureCommunications2014	1	(27)
GelabertBioRxiv2023	1	(29)
GelabertSciRep2022	9	(28)
HarneyCheronetGenomeResearch2021	2	(36)
HarneyMayNatureCommunications2018	11	(35)
HofmanovaPNAS2016	2	(37)
JensenSchroederNatureCommunications2019	1	(41)
LazaridisAlpaslanRoodenbergScience2022	45	(49)
LazaridisNature2016	8	(47)
LipsonNature2017	12	(51)
MarcusNatureCommunications2020	3	(59)
MartinianoPLoSGenetics2017	4	(60)
MathiesonNature2018	15	(63)
MattilaCommBio2023	5	(64)
NarasimhanPattersonScience2019	1	(69)
NovakPLoSOne2021	27	(70)
OlaldeNature2018	16	(72)
OlaldeScience2019	2	(73)
PapacScienceAdvances2021	26	(74)
PattersonNature2021	9	(75)
PenskeHaakNature2023	46	(76)
RivollatNature2023	3	(83)
RivollatScienceAdvance2020	24	(82)
SanchezQuintoPNAS2019	2	(95)
ScheibAnnHumBio2019	1	(86)
SimoesNature2023	1	(90)
SkourtaniotiCell2020	10	(92)
VillalbaMoucoCurrentBiology2019	2	(97)
WangNatureCommunications2019	4	(99)

Table S5: References for samples used in the 4500-3500 BCE dataset

Study (Abbreviation in AADR)	Samples	Reference
AgranatTamirWaldmanCell2020	17	(1)
AllentoftNature2015	5	(2)
AmorimNatureCommunications2018	1	(4)
AntonioGaoMootsScience2019	1	(5)
ArianoCell2022	2	(6)
BlocherPNAS2023	32	(11)
ClementeCell2021	6	(19)
DamgaardScience2018	3	(20)
FernandesNatureEcologyEvolution2020	19	(23)
FernandesScientificReports2018	5	(22)
FreilichPinhasiScientificReports2021	6	(25)
FurtwanglerNatureCommunications2020	26	(26)
GelabertSciRep2022	1	(28)
GonzalesFortesProcRoyalSocB2019	1	(33)
HaberAJHG2017	4	(34)
HarneyCheronetGenomeResearch2021	1	(36)
IngmanStockhammerPLoS2021	8	(40)
KrzewinskaScienceAdvances2018	6	(44)
LazaridisAlpaslanRoodenbergScience2022	63	(49)
LazaridisNature2016	3	(47)
LazaridisNature2017	4	(48)
LinderholmNatureScientificReports2020	4	(50)
MarcusNatureCommunications2020	19	(59)
MartinianoPLoSGenetics2017	2	(60)
MathiesonNature2015	20	(62)
MathiesonNature2018	5	(63)
MittnikScience2019	44	(67)
MootsNatEcolEvol2023	2	(68)
NarasimhanPattersonScience2019	92	(69)
OlaldeNature2018	148	(72)
OlaldeScience2019	24	(73)
PapacScienceAdvances2021	95	(74)
PattersonNature2021	61	(75)
PenskeSciRep2024	40	(77)
RohlandMallickGenomeResearch2022	1	(84)
SeguinOrlandoCurrBio2021	3	(89)
SkourtaniotiCell2020	20	(92)
SkourtaniotiRingbauerNatureEcologyEvolution2023	27	(93)
VillalbaMoucoSciAdv2021	70	(98)
WangNatureCommunications2019	7	(99)
ZegaracScientificReports2021	18	(102)

Table S6: References for samples used in the 2500-1500 BCE dataset

References

[1] Agranat-Tamir, L. et al. (2020). The genomic history of the bronze age southern levant. *Cell*, 181(5):1146–1157.

[2] Allentoft, M. E. et al. (2015). Population genomics of bronze age eurasia. *Nature*, 522(7555):167–172.

[3] Allentoft, M. E. et al. (2024). Population genomics of post-glacial western eurasia. *Nature*, 625:301–311.

[4] Amorim, C. E. G. et al. (2018). Understanding 6th-century barbarian social organization and migration through paleogenomics. *Nat Commun*, 9(1):3547.

[5] Antonio, M. L. et al. (2019). Ancient rome: A genetic crossroads of europe and the mediterranean. *Science*, 366(6466):708–714.

[6] Ariano, B. et al. (2022). Ancient maltese genomes and the genetic geography of neolithic europe. *Curr. Biol.*, 32:2668–2680.e6.

[7] Barata, J. C. A. and Hussein, M. S. (2012). The moore–penrose pseudoinverse: A tutorial review of the theory. *Braz. J. Phys.*, 42:146–165.

[8] Bartels, R. H. and Stewart, G. W. (1972). Solution of the matrix equation ax + xb = c. *Communications of the ACM*, 15(9):820–826.

[9] Baumdicker, F. et al. (2022). Efficient ancestry and mutation simulation with msprime 1.0. *Genetics*, 220:iyab229.

[10] Berv, J. et al. (2021). Genomic phylogeography of the white-crowned manakin *Pseudopipra pipra* (aves: Pipridae) illuminates a continental-scale radiation out of the andes. *Mol. Phylogenet. Evol.*, 159:107111.

[11] Blöcher, J. et al. (2023). Descent, marriage, and residence practices of a 3,800-year-old pastoral community in central eurasia. *Proc. Natl Acad. Sci. USA*, 120:e2303574120.

[12] Brace, S. et al. (2019). Ancient genomes indicate population replacement in early neolithic britain. *Nat Ecol Evol*, 3(5):765–771.

[13] Brunel, S. et al. (2020). Ancient genomes from present-day france unveil 7,000 years of its demographic history. *Proc. Natl Acad. Sci. USA*, 117(23):12791–12798.

[14] Byrd, R. H., Lu, P., Nocedal, J., and Zhu, C. (1995). A limited memory algorithm for bound constrained optimization. *SIAM J. Sci. Comput.*, 16:1190–1208.

[15] Cassidy, L. M. et al. (2016). Neolithic and bronze age migration to ireland and establishment of the insular atlantic genome. *Proc. Natl Acad. Sci. USA*, 113(2):368–373.

[16] Cassidy, L. M. et al. (2020). A dynastic elite in monumental neolithic society. *Nature*, 582(7812):384–388.

[17] Caughman, J. S. and Veerman, J. J. P. (2006). Kernels of directed graph laplacians. *Electron. J. Combin.*, 13:R39.

[18] Childebayeva, A. et al. (2022). Population genetics and signatures of selection in early neolithic european farmers. *Mol Biol Evol*, 39(6):msac108.

[19] Clemente, F. et al. (2021). The genomic history of the aegean palatial civilizations. *Cell*, 184(10):2565–2586.

[20] Damgaard, P. B. et al. (2018). 137 ancient human genomes from across the eurasian steppes. *Nature*, 557(7705):369–374.

[21] Feldman, M. et al. (2019). Late pleistocene human genome suggests a local origin for the first farmers of central anatolia. *Nat Commun*, 10(1):1218.

[22] Fernandes, D. M. et al. (2018). A genomic neolithic time transect of hunter-farmer admixture in central poland. *Sci Rep*, 8(1):14879.

[23] Fernandes, D. M. et al. (2020). The spread of steppe and iranian-related ancestry in the islands of the western mediterranean. *Nat Ecol Evol*, 4(3):334–345.

[24] Fowler, C. et al. (2022). A high-resolution picture of kinship practices in an early neolithic tomb. *Nature*, 601(7894):584–587.

[25] Freilich, S. et al. (2021). Reconstructing genetic histories and social organisation in neolithic and bronze age croatia. *Sci Rep*, 11(1):16729.

[26] Furtwängler, A. et al. (2020). Comparison of target enrichment strategies for ancient pathogen dna. *Biotechniques*, 69(6):455–459.

[27] Gamba, C. et al. (2014). Genome flux and stasis in a five millennium transect of european prehistory. *Nat Commun*, 5(1):5257.

[28] Gelabert, P. et al. (2022). Genomes from verteba cave suggest diversity within the trypillians in ukraine. *Sci Rep*, 12(1):7242.

[29] Gelabert, P. et al. (2023). Social and genetic diversity among the first farmers of central europe. *bioRxiv*.

[30] Geraldes, A. et al. (2014). Landscape genomics of *Populus trichocarpa*: the role of hybridization, limited gene flow, and natural selection in shaping patterns of population structure. *Evolution*, 68:3260–3280.

[31] Gokhman, D. et al. (2020). Differential dna methylation of vocal and facial anatomy genes in modern humans. *Nat Commun*, 11(1):1189.

[32] González-Fortes, G. et al. (2017). Paleogenomic evidence for multi-generational mixing between neolithic farmers and mesolithic hunter-gatherers in the lower danube basin. *Curr Biol*, 27(12):1801–1810.

[33] González-Fortes, G. et al. (2019). A western route of prehistoric human migration from africa into the iberian peninsula. *Proc Biol Sci*, 286(1895):20182288.

[34] Haber, M. et al. (2017). Continuity and admixture in the last five millennia of levantine history from ancient canaanite and present-day lebanese genome sequences. *Am J Hum Genet*, 101(2):274–282.

[35] Harney, E. et al. (2018). Ancient dna from chalcolithic israel reveals the role of population mixture in cultural transformation. *Nat Commun*, 9(1):3336.

[36] Harney, E. et al. (2021). A minimally destructive protocol for dna extraction from ancient teeth. *Genome Res*, 31(3):472–483.

[37] Hofmanová, Z. et al. (2016). Early farmers from across europe directly descended from neolithic aegeans. *Proc Natl Acad Sci USA*, 113(25):6886–6891.

[38] Immel, A. et al. (2020). Gene-flow from steppe individuals into cucuteni-trypillia associated populations indicates long-standing contacts and gradual admixture. *Sci Rep*, 10:4253.

[39] Immel, A. et al. (2021). Genome-wide study of a neolithic wartberg grave community reveals distinct hla variation and hunter-gatherer ancestry. *Commun Biol*, 113(1):113.

[40] Ingman, T. et al. (2021). Turkey during the 2nd millennium bc: Integration of isotopic and genomic evidence. *PLoS One*, 16(6):e0241883.

[41] Jensen, T. Z. T. et al. (2019). A 5700-year-old human genome and oral microbiome from chewed birch pitch. *Nat Commun*, 10(1):5520.

[42] Jeong, C. et al. (2019). The genetic history of admixture across inner eurasia. *Nat Ecol Evol*, 3(6):966–976.

[43] Koptekin, D. et al. (2023). Spatial and temporal heterogeneity in human mobility patterns in holocene southwest asia and the east mediterranean. *Curr Biol*, 33(1):41–57.

[44] Krzewińska, M. et al. (2018). Ancient genomes suggest the eastern pontic-caspian steppe as the source of western iron age nomads. *Sci Adv*, 4(10):eaat4457.

[45] Laub, A. J. (2005). *Matrix Analysis for Scientists and Engineers*. SIAM.

[46] Lazaridis, I. et al. (2014). Ancient human genomes suggest three ancestral populations for present-day europeans. *Nature*, 513(7518):409–413.

[47] Lazaridis, I. et al. (2016). Genomic insights into the origin of farming in the ancient near east. *Nature*, 536(7617):419–424.

[48] Lazaridis, I. et al. (2017). Genetic origins of the minoans and mycenaeans. *Nature*, 548(7666):214–218.

[49] Lazaridis, I. et al. (2022). The genetic history of the southern arc: A bridge between west asia and europe. *Science*, 377(6609):eabm4247.

[50] Linderholm, A. et al. (2020). Corded ware cultural complexity uncovered using genomic and isotopic analysis from south-eastern poland. *Sci Rep*, 10(1):6885.

[51] Lipson, M. et al. (2017). Parallel palaeogenomic transects reveal complex genetic history of early european farmers. *Nature*, 551(7680):368–372.

[52] Lundgren, E. and Ralph, P. L. (2019). Are populations like a circuit? comparing isolation by resistance to a new coalescent-based method. *Mol. Ecol. Resour.*, 19:1388–1406.

[53] Maier, R. et al. (2023). On the limits of fitting complex models of population history to f-statistics. *eLife*, 12:e85492.

[54] Mallick, S. et al. (2024). The allen ancient dna resource (aadr): a curated compendium of ancient human genomes. *Sci. Data*, 11:182.

[55] Mallick, S. and Reich, D. (2023). The allen ancient dna resource (aadr): A curated compendium of ancient human genomes. https://doi.org/10.7910/DVN/FFIDCW.

[56] Malmström, H. et al. (2019). The genomic ancestry of the scandinavian battle axe culture people and their relation to the broader corded ware horizon. *Proc Biol Sci*, 286(1912):20191528.

[57] Marchi, N. et al. (2022). The genomic origins of the world's first farmers. *Cell*, 185:1842–1859.e18.

[58] Marcus, J., Ha, W., Barber, R. F., and Novembre, J. (2021). Fast and flexible estimation of effective migration surfaces. *eLife*, 10:e61927.

[59] Marcus, J. H. et al. (2020). Genetic history from the middle neolithic to present on the mediterranean island of sardinia. *Nat Commun*, 11(1):939.

[60] Martiniano, R. et al. (2017). The population genomics of archaeological transition in west iberia: Investigation of ancient substructure using imputation and haplotype-based methods. *PLoS Genet*, 13(7):e1006852.

[61] Maróti, Z. et al. (2022). The genetic origin of huns, avars, and conquering hungarians. *Curr Biol*, 32(13):2858–2870.

[62] Mathieson, I. et al. (2015). Genome-wide patterns of selection in 230 ancient eurasians. *Nature*, 528(7583):499–503.

[63] Mathieson, I. et al. (2018). The genomic history of southeastern europe. *Nature*, 555(7695):197–203.

[64] Mattila, T. M. et al. (2023). Genetic continuity, isolation, and gene flow in stone age central and eastern europe. *Commun Biol*, 6:793.

[65] McVean, G. (2009). A genealogical interpretation of principal components analysis. *PLoS Genetics*, 5:e1000686.

[66] Meyer, C. D. (1973). Generalized inversion of modified matrices. *SIAM J. Appl. Math.*, 24:315–323.

[67] Mittnik, A. et al. (2019). Kinship-based social inequality in bronze age europe. *Science*, 366(6466):731–734.

[68] Moots, H. M. et al. (2023). A genetic history of continuity and mobility in the iron age central mediterranean. *Nat Ecol Evol*, 7:1515–1524.

[69] Narasimhan, V. M. et al. (2019). The formation of human populations in south and asia. *Science*, 365(6457):eaat7487.

[70] Novak, M. et al. (2021). Genome-wide analysis of nearly all the victims of a 6200-year-old massacre. *PLoS One*, 16(3):e0247332.

[71] Olalde, I. et al. (2015). A common genetic origin for early farmers from mediterranean cardial and european lbk cultures. *Mol Biol Evol*, 32(12):3132–3142.

[72] Olalde, I. et al. (2018). The beaker phenomenon and the genomic transformation of northwest europe. *Nature*, 555(7695):190–196.

[73] Olalde, I. et al. (2019). The genomic history of the iberian peninsula over the past 8000 years. *Science*, 363(6432):1230–1234.

[74] Papac, L. et al. (2021). Dynamic changes in genomic and social structures in third millennium bce central europe. *Sci Adv*, 7(35):eabi6941.

[75] Patterson, N. et al. (2022). Large-scale migration into britain during the middle to late bronze age. *Nature*, 601(7894):588–594.

[76] Penske, S. et al. (2023). Early contact between late farming and pastoralist societies in southeastern europe. *Nature*, 620:358–365.

[77] Penske, S. et al. (2024). Kinship practices at the early bronze age site of leubingen in central germany. *Sci Rep*, 14:3871.

[78] Petkova, D., Novembre, J., and Stephens, M. (2016). Visualizing spatial population structure with estimated effective migration surfaces. *Nature Genetics*, 48:94–100.

[79] Posth, C. et al. (2023). Palaeogenomics of upper palaeolithic to neolithic european hunter-gatherers. *Nature*, 615:117–126.

[80] Purcell, S. et al. (2007). Plink: a tool set for whole-genome association and population-based linkage analyses. *Am. J. Hum. Genet.*, 81:559–575.

[81] Ramachandran, S. et al. (2005). Support from the relationship of genetic and geographic distance in human populations for a serial founder effect originating in africa. *Proc. Natl Acad. Sci. USA*, 102:15942–15947.

[82] Rivollat, M. et al. (2020). Ancient genome-wide dna from france highlights the complexity of interactions between mesolithic hunter-gatherers and neolithic farmers. *Sci Adv*, 6(22):eaaz5344.

[83] Rivollat, M. et al. (2023). Extensive pedigrees reveal the social organization of a neolithic community. *Nature*, 620:600–606.

[84] Rohland, N. et al. (2015). Partial uracil-dna-glycosylase treatment for screening of ancient dna. *Philos Trans R Soc Lond B Biol Sci*, 370(1660):p20130624.

[85] Saupe, T. et al. (2021). Ancient genomes reveal structural shifts after the arrival of steppe-related ancestry in the italian peninsula. *Curr Biol*, 31(12):2576–2591.

[86] Scheib, C. L. et al. (2019). East anglian early neolithic monument burial linked to contemporary megaliths. *Ann Hum Biol*, 46(2):145–149.

[87] Schroeder, H. et al. (2019). Unraveling ancestry, kinship, and violence in a late neolithic mass grave. *Proc Natl Acad Sci USA*, 116(22):10705–10710.

[88] Schweizer, R. M. et al. (2016). Genetic subdivision and candidate genes under selection in north american grey wolves. *Molecular Ecology*, 25:380–402.

[89] Seguin-Orlando, A. et al. (2021). Heterogeneous hunter-gatherer and steppe-related ancestries in late neolithic and bell beaker genomes from present-day france. *Curr Biol*, 31(5):1072–1083.

[90] Simoes, L. et al. (2023). Northwest african neolithic initiated by migrants from iberia and levant. *Nature*, 618:550–556.

[91] Simoes, L. et al. (2024). Genomic ancestry and social dynamics of the last hunter-gatherers of atlantic france. *PNAS*, 121(10):e2310545121.

[92] Skourtanioti, E. et al. (2020). Genomic history of neolithic to bronze age anatolia, northern levant, and southern caucasus. *Cell*, 181(5):1158–1175.

[93] Skourtanioti, E. et al. (2023). Ancient dna reveals admixture history and endogamy in the prehistoric aegean. *Nat Ecol Evol*, 7:290–303.

[94] Strobeck, C. (1987). Average number of nucleotide differences in a sample from a single subpopulation: a test for population subdivision. *Genetics*, 117:149–153.

[95] Sánchez-Quinto, F. et al. (2019). Megalithic tombs in western and northern neolithic europe were linked to a kindred society. *Proc Natl Acad Sci USA*, 116(19):9469–9474.

[96] Valdiosera, C. et al. (2018). Four millennia of iberian biomolecular prehistory illustrate the impact of prehistoric migrations at the far end of eurasia. *Proc Natl Acad Sci USA*, 115(13):3428–3433.

[97] Villalba-Mouco, V. et al. (2019). Survival of late pleistocene hunter-gatherer ancestry in the iberian peninsula. *Curr Biol*, 29(7):1169–1177.

[98] Villalba-Mouco, V. et al. (2021). Genomic transformation and social organization during the copper age-bronze age transition in southern iberia. *Sci Adv*, 7(47):eabi7038.

[99] Wang, C. C. et al. (2019). Ancient human genome-wide data from a 3000-year interval in the caucasus corresponds with eco-geographic regions. *Nat Commun*, 10(1):590.

[100] Wang, K. et al. (2023). High-coverage genome of the tyrolean iceman reveals unusually high anatolian farmer ancestry. *Cell Genomics*, 3:100377.

[101] Yu, H. et al. (2022). Genomic and dietary discontinuities during the mesolithic and neolithic in sicily. *iScience*, 25(5):104244.

[102] Žegarac, A. et al. (2021). Ancient genomes provide insights into family structure and the heredity of social status in the early bronze age of southeastern europe. *Sci Rep*, 11(1):10072.

Figure S1: FRAME fit to equilibrium migration simulations with different patterns and sampling schemes (Difference graph)

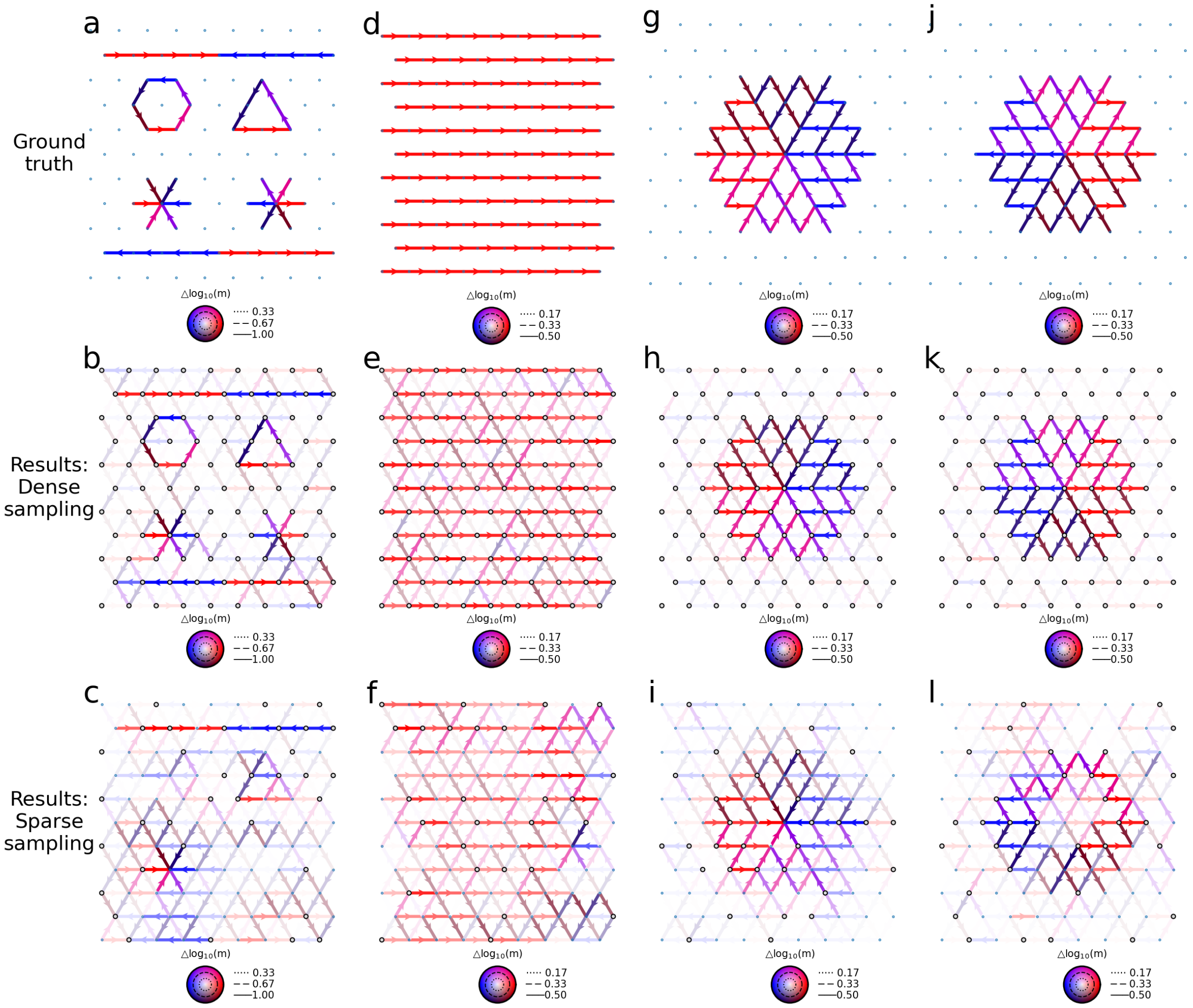

(**a–l**) Difference graphs corresponding to full graphs in Figure 3.

Figure S2: FRAME fit to simulations of large scale directional flow with equal coalescence rates

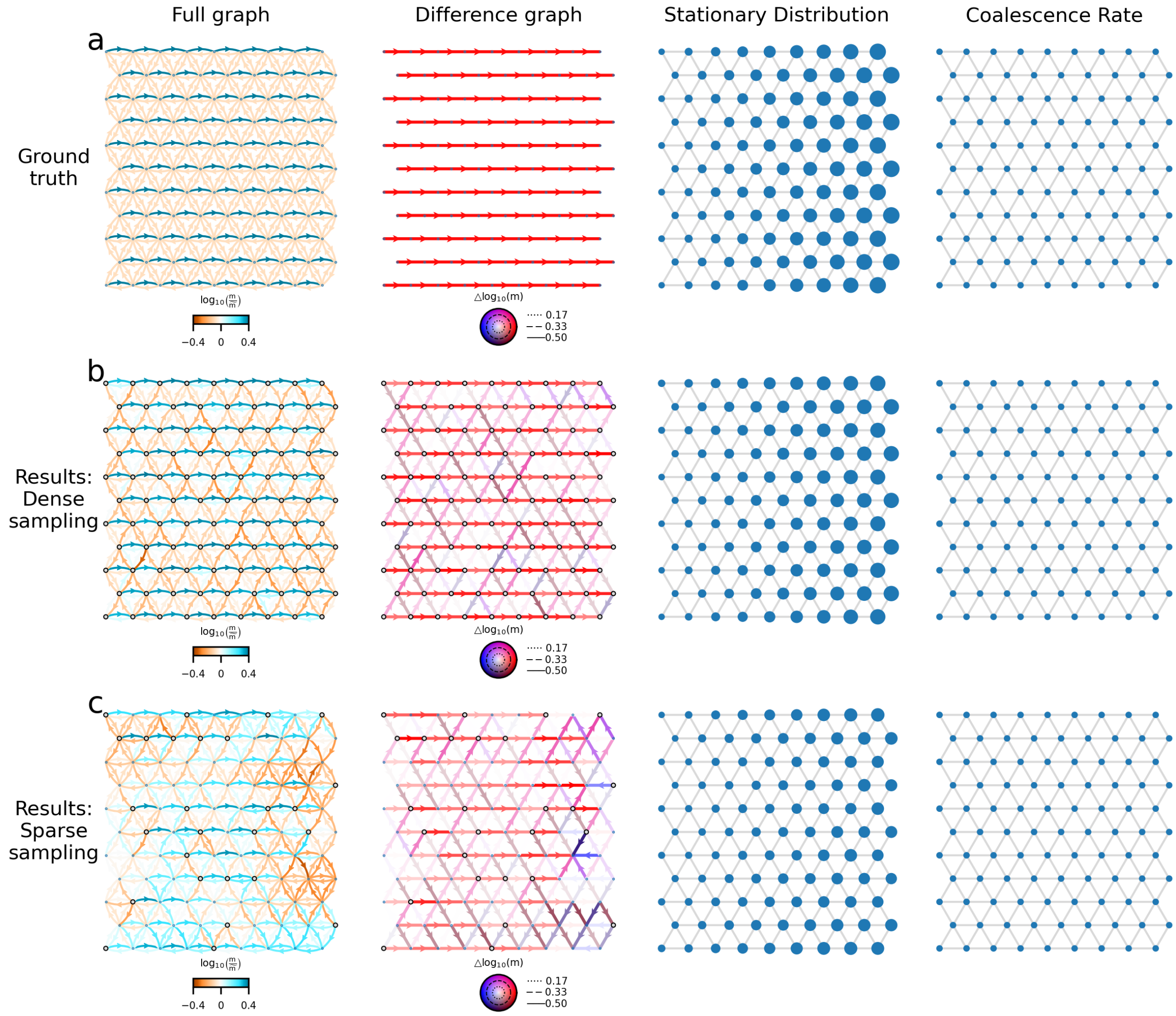

(a-c) Ground truth and FRAME fit to simulations of large scale directional flow with equal coalescence rates, varying the sampling design

Figure S3: FRAME fit to simulations of large scale directional flow with coalescence rates inversely proportional to stationary distribution

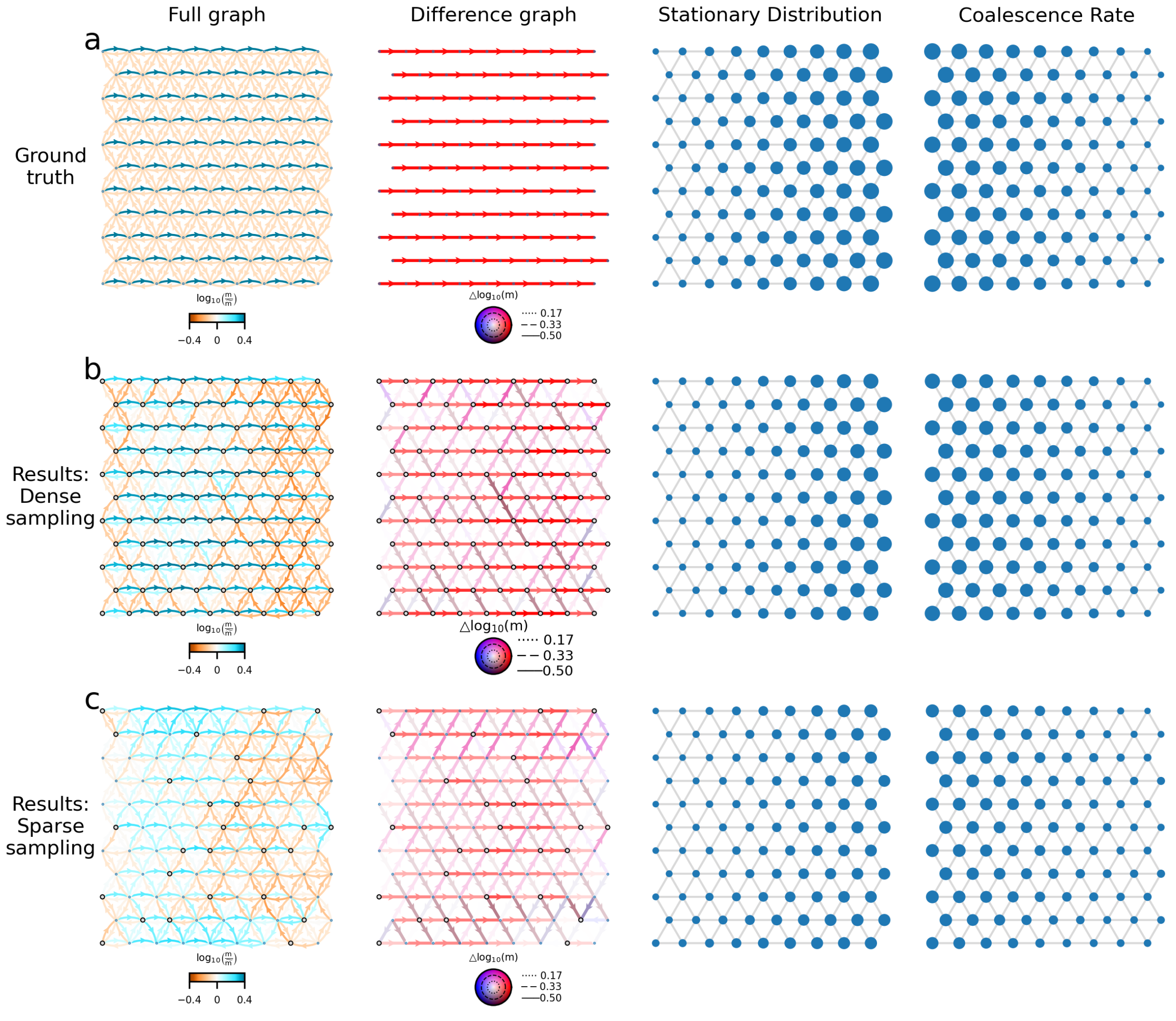

(a-c) Ground truth and FRAME fit to simulations of large scale directional flow with coalescence rates inversely proportional to stationary distribution, varying the sampling design

Figure S4: Comparison of FRAME with Lundgren and Ralph (2019) on the poplars dataset

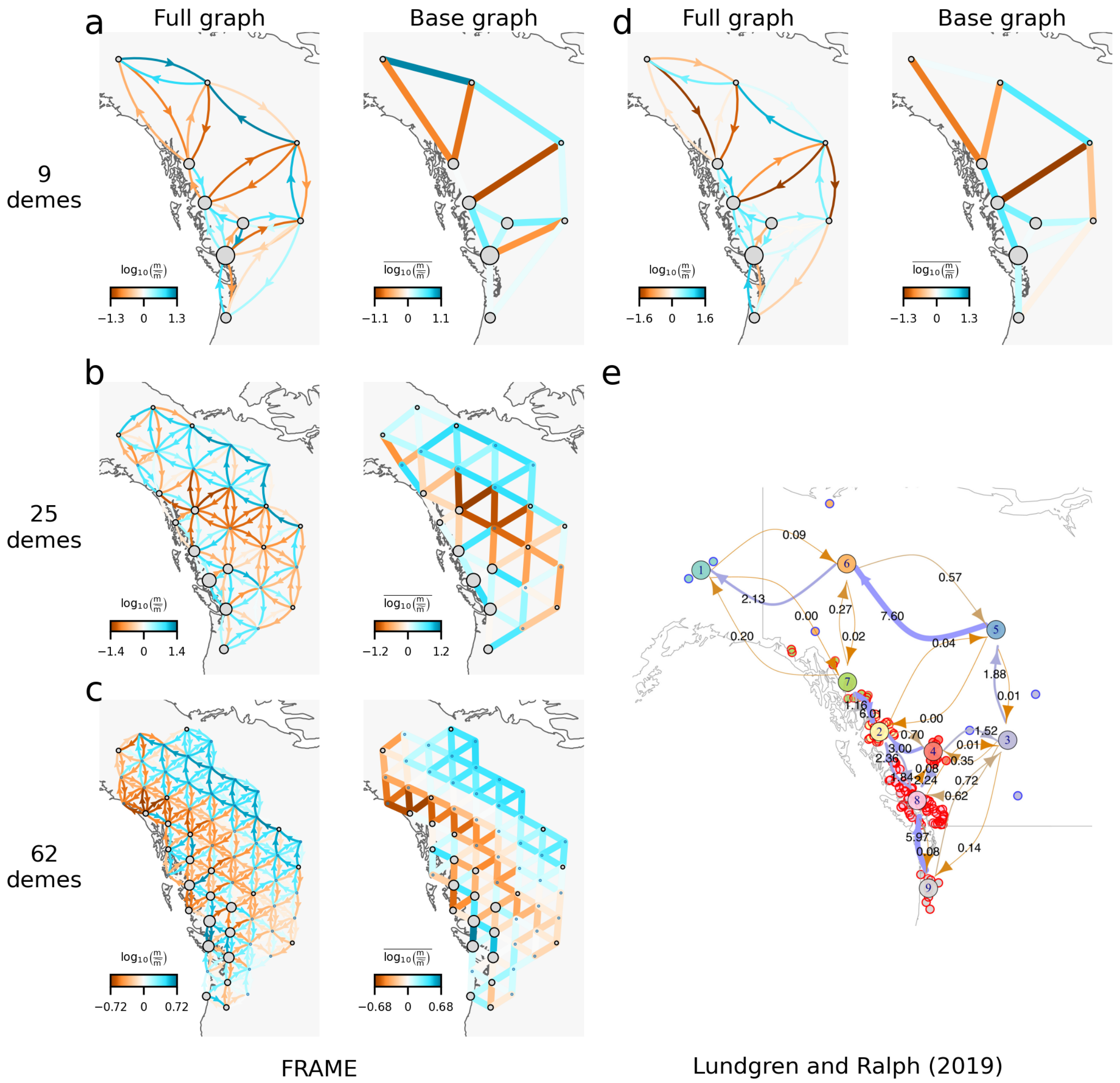

(a–c) FRAME analysis of the *Populus trichocarpa/balsamifera* datasets dataset at different resolutions. **(d)** Lundgren and Ralph (2019) results visualized in FRAME's representation, with zero edges replaced by a minimal migration rate of 0.01. **(e)** Lundgren and Ralph (2019) results in their original representation.

Figure S5: Comparison of the 7000-3500 BCE dataset with the 4500-3500 BCE dataset

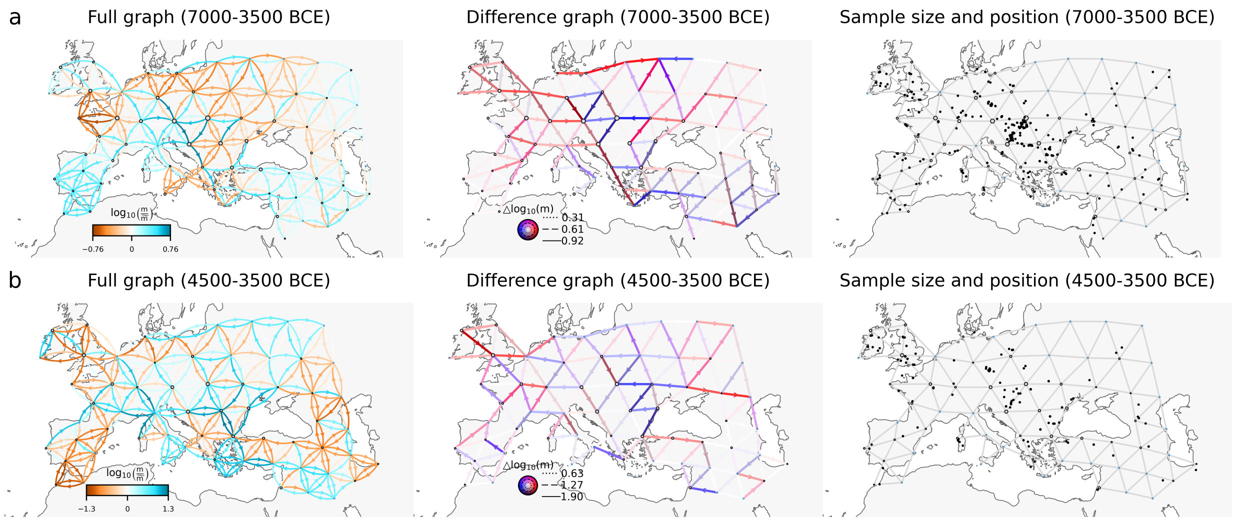

(a) FRAME analysis of the 7000-3500 BCE dataset. **(b)**FRAME analysis of the 4500-3500 dataset. Although FRAME analysis of this dataset shares some asymmetric signals with the FRAME analysis of the 7000-3500 BCE dataset, it detects fewer patterns consistent with our current knowledge Neolithic expansion. This discrepancy is likely due to the smaller sample size (383 individuals versus 975 in the broader window) and time-varying gene flow dynamics during these periods. For instance, the 4500-3500 BCE dataset lacks samples from southern France, and we observe different signals along the northern Mediterranean coast. Similarly, the significant reduction of number of samples from Germany probably leads to notable differences in the inferred migration patterns in that region compared to the 7000-3500 BCE dataset.

Figure S6: Comparison of the 3500-1500 BCE dataset with the 2500-1500 BCE dataset

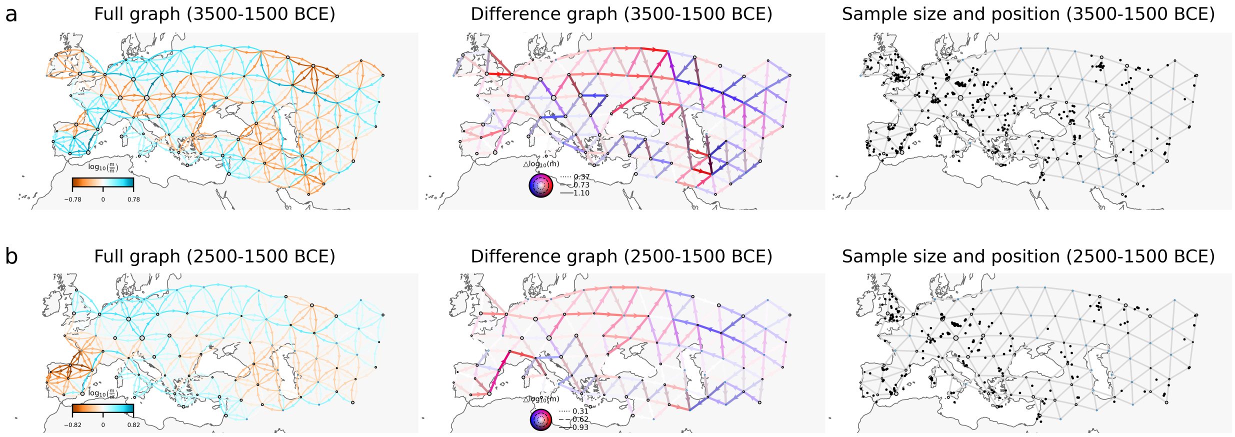

(a) FRAME analysis of the 3500-1500 BCE dataset. **(b)**FRAME analysis of the 2500-1500 dataset. Compared with Figure S5, FRAME analysis of the 2500-1500 BCE dataset shows more similarity with FRAME analysis of the $3500 - 1500$ BCE dataset. The asymmetries plausibly reflecting gene flow out of the Steppe region is a shared signal in both analyses. This similarity is likely due to relative large sample sizes of the $2500 - 1500$ BCE dataset (916 individuals versus 1361 individuals in the $3500 - 1500$ BCE dataset).

Figure S7: Graph Representations

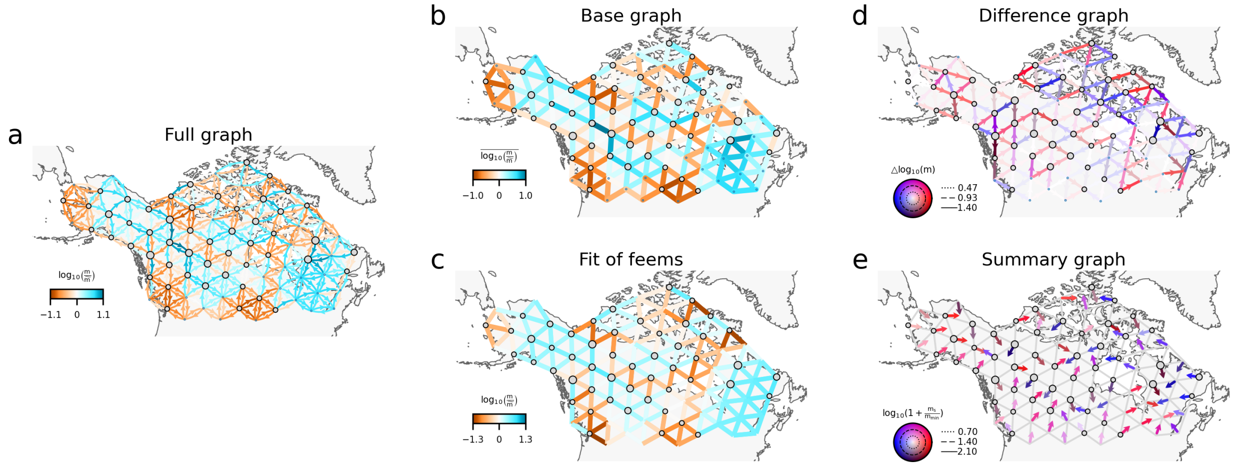

(a) Full graph representation uses inferred migration rates as edge weights in a directed graph, depicting the complete migration scenario. **(b)** For a pair of connected demes (A, B), the base graph representation assigns the average log-scaled migration rate $\frac{1}{2}(\log m_{AB} + \log m_{BA})$ to the undirected edge AB. Qualitatively, it resembles FEEMS and EEMS and can be interpreted as a connectivity graph. **(c)** For a pair of connected demes (A, B) where $m_{AB} > m_{BA}$, the difference graph representation assigns the log-scale difference $\log m_{AB} - \log m_{BA}$ to the directed edge AB. This representation complements the base graph. **(d)** Summary graph representation sums all outgoing edge vectors at each node, producing a migration vector field.

Figure S8: FRAME fit to the wolf dataset (full version)

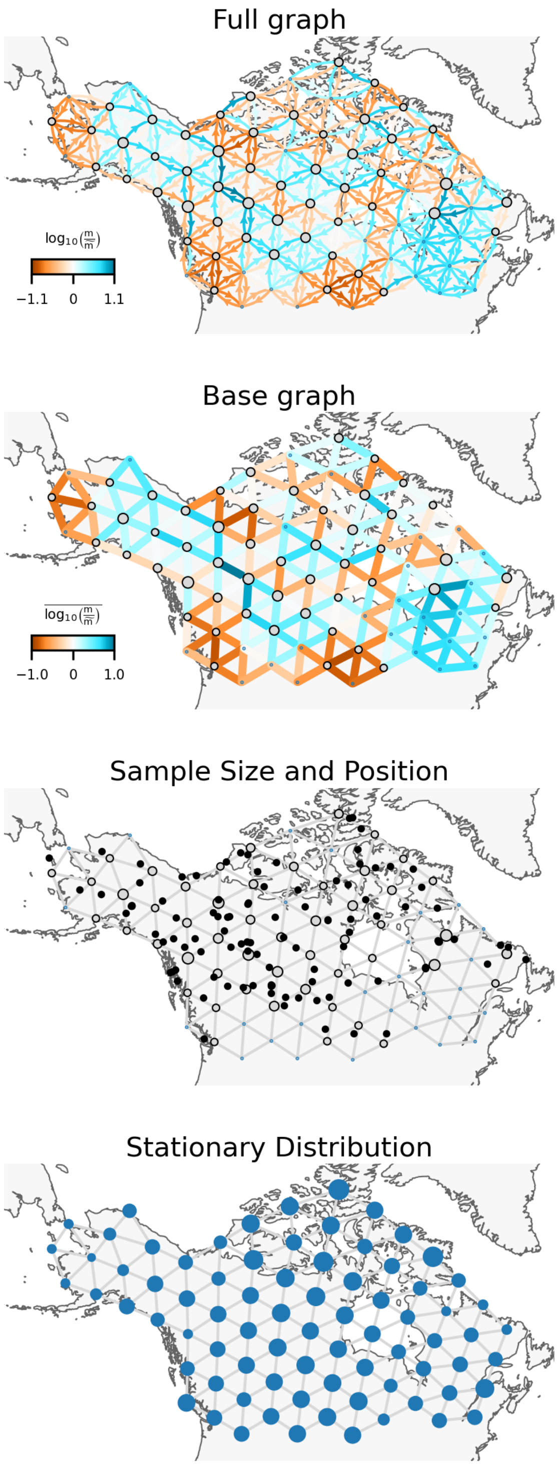

The first two rows presents all four representations of the FRAME fit to the wolf dataset. The third and fourth row displays the corresponding dataset attributes.

Figure S9: FRAME fit to the poplars dataset (full version)

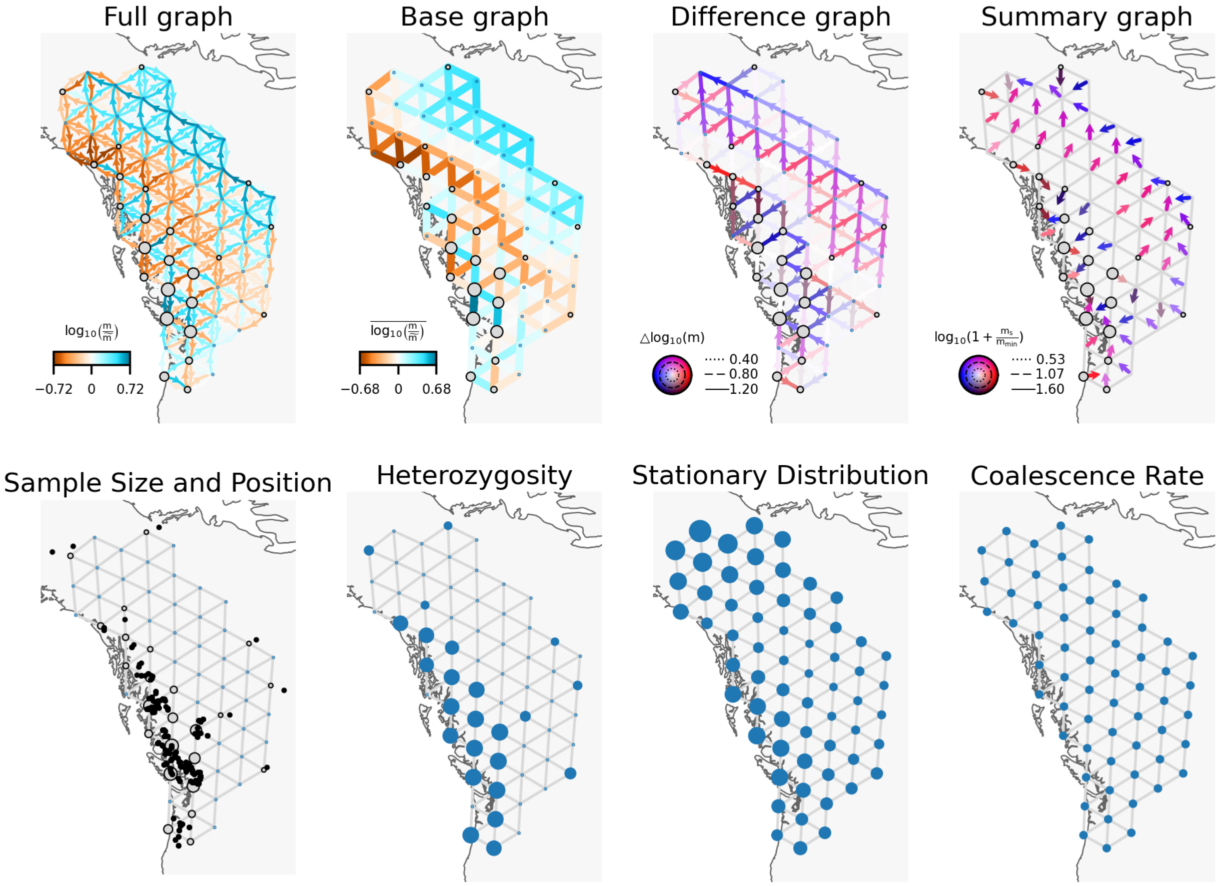

The first row presents all four representations of the FRAME fit to the poplars dataset. The second row displays the corresponding dataset attributes.

Figure S10: FRAME fit to the manakin dataset (full version)

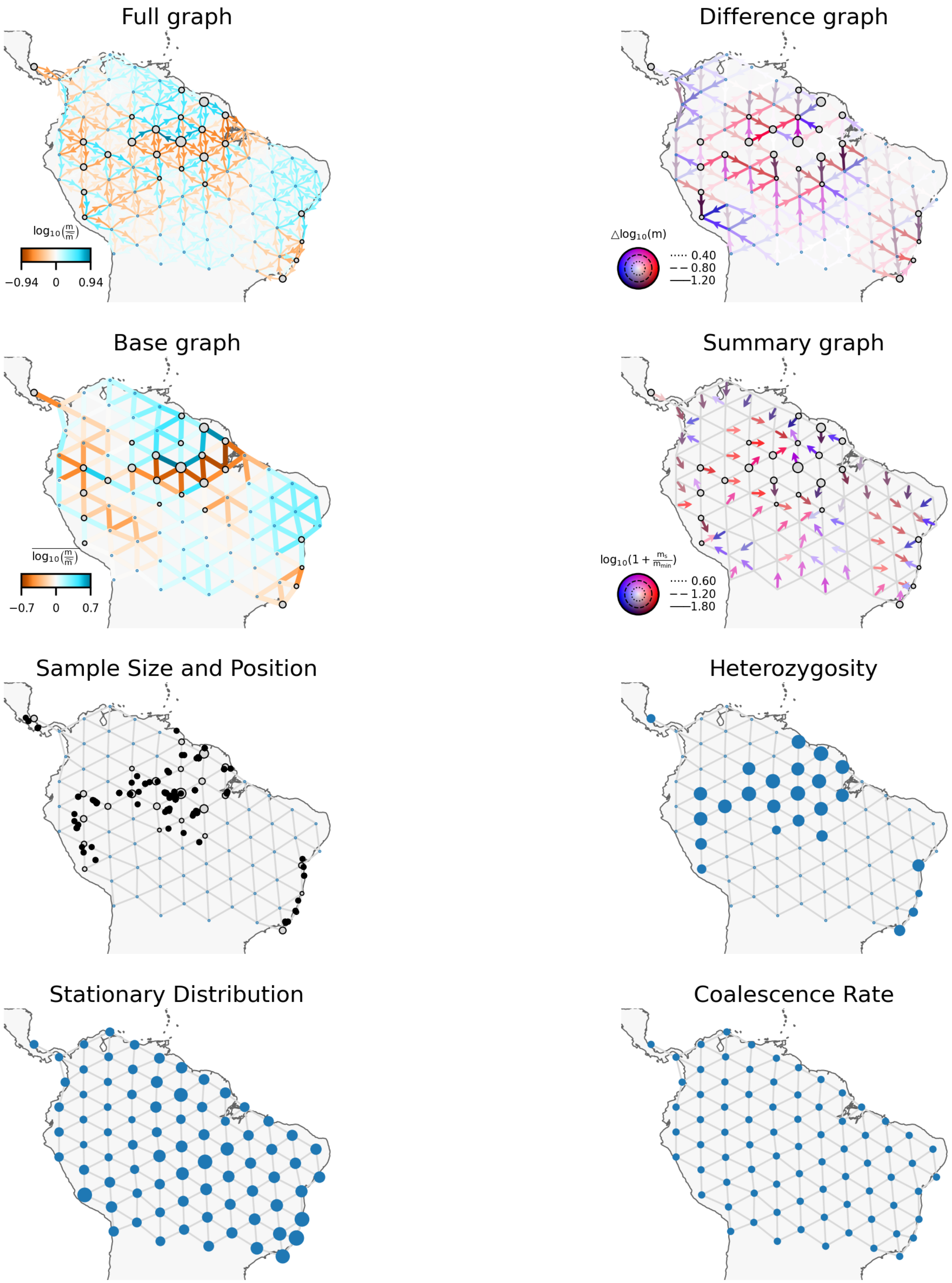

The first two rows presents all four representations of the FRAME fit to the manakin dataset. The third and fourth row displays the corresponding dataset attributes.

Figure S11: FRAME fit to the 7000-3500 BCE aDNA dataset (full version)

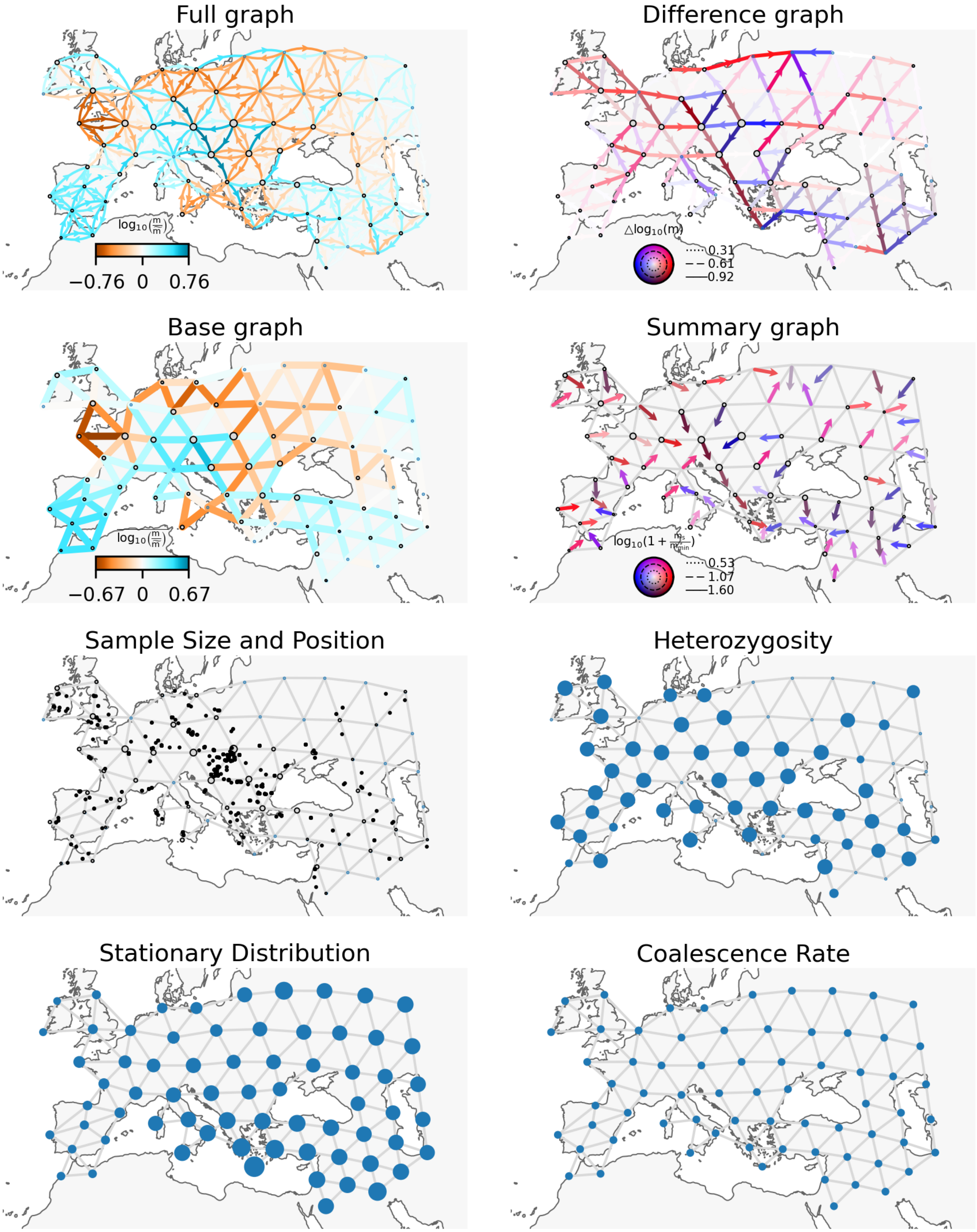

The first two rows presents all four representations of the FRAME fit to the 7000-3500 BCE aDNA dataset. The third and fourth row displays the corresponding dataset attributes.

Figure S12: FRAME fit to the 3500-1500 BCE aDNA dataset (full version)

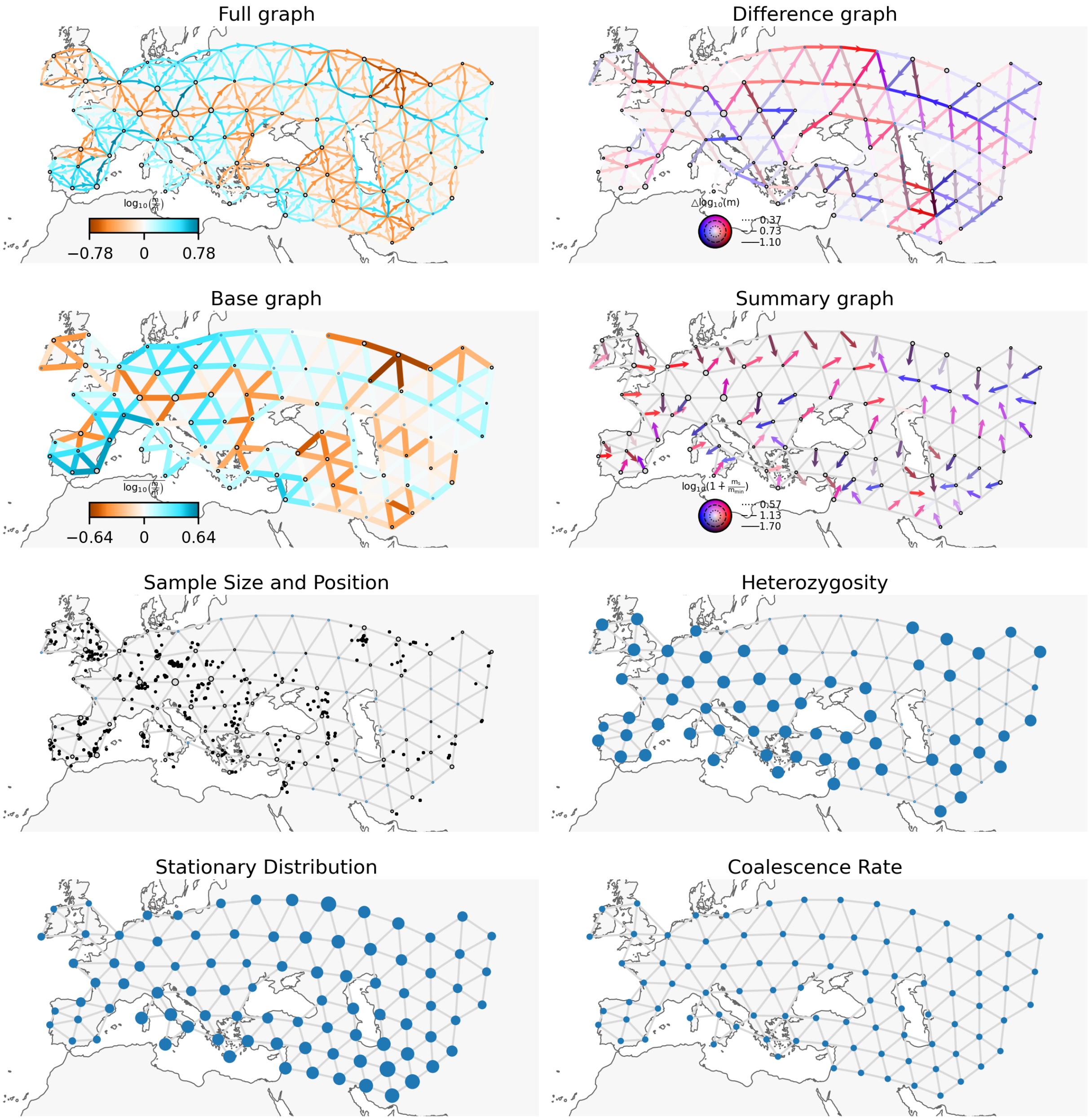

The first two rows presents all four representations of the FRAME fit to the 3500-1500 BCE aDNA dataset. The third and fourth row displays the corresponding dataset attributes.

Figure S13: FRAME fit to the 4500-3500 BCE aDNA dataset (full version)

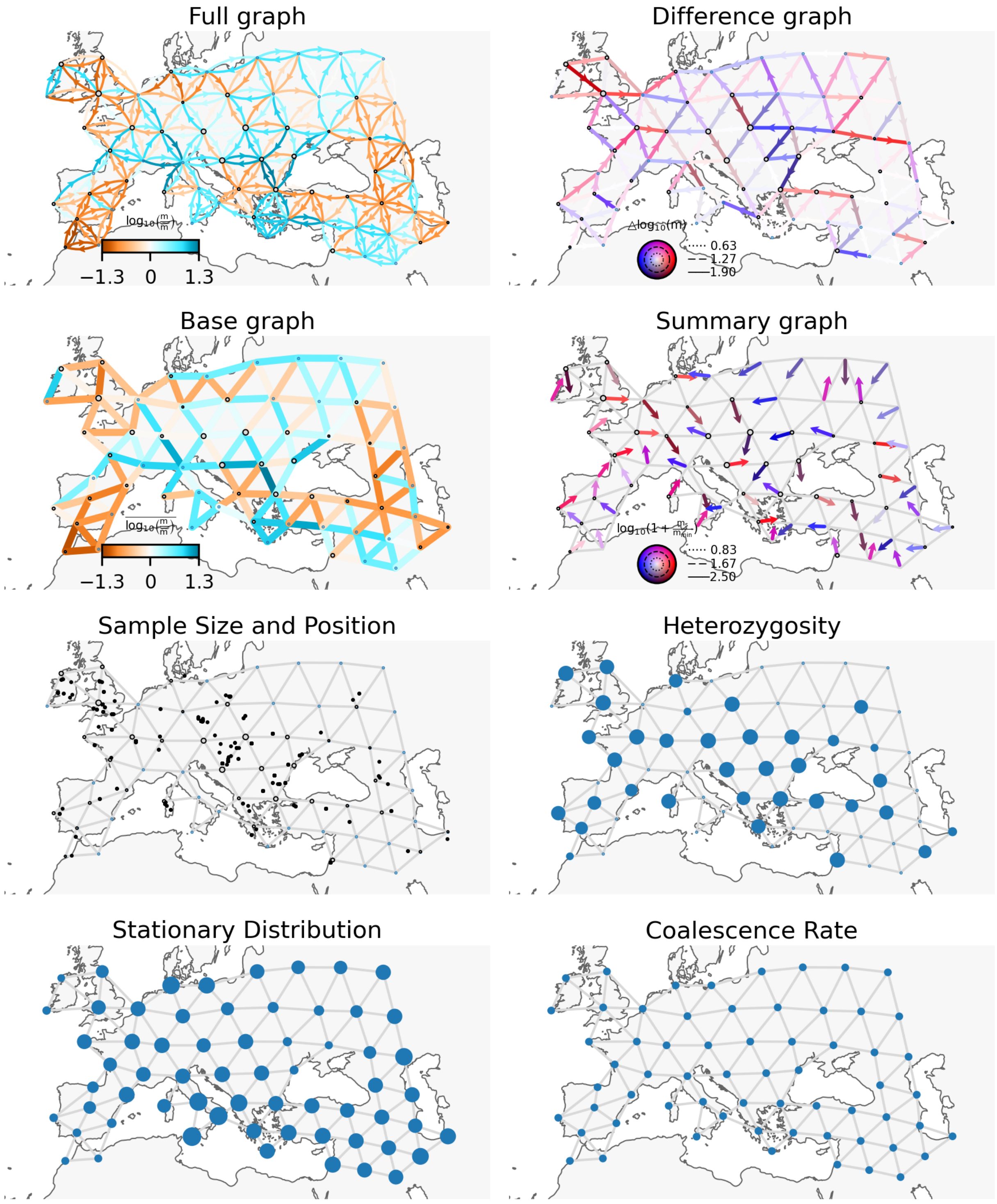

The first two rows presents all four representations of the FRAME fit to the 4500-3500 BCE aDNA dataset. The third and fourth row displays the corresponding dataset attributes.

Figure S14: FRAME fit to the 2500-1500 BCE aDNA dataset (full version)

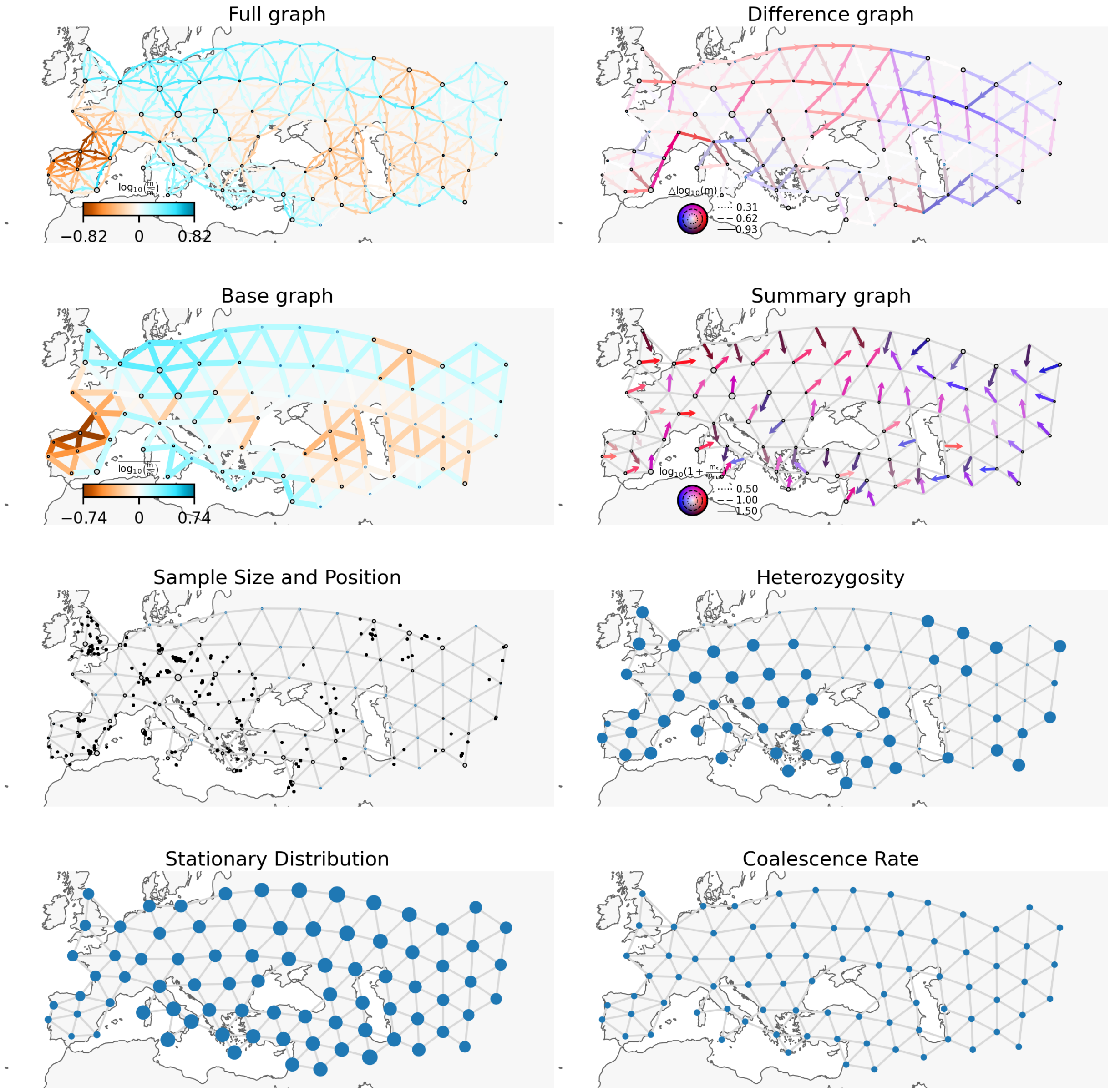

The first two rows presents all four representations of the FRAME fit to the 2500-1500 BCE aDNA dataset. The third and fourth row displays the corresponding dataset attributes.
